# Periodic Oxaliplatin Administration in Synergy with PER2‐Mediated *PCNA* Transcription Repression Promotes Chronochemotherapeutic Efficacy of OSCC

**DOI:** 10.1002/advs.201900667

**Published:** 2019-09-08

**Authors:** Qingming Tang, Mengru Xie, Shaoling Yu, Xin Zhou, Yanling Xie, Guangjin Chen, Fengyuan Guo, Lili Chen

**Affiliations:** ^1^ Department of Stomatology Union Hospital Tongji Medical College Huazhong University of Science and Technology Wuhan 430022 China

**Keywords:** chronochemotherapeutic strategy, circadian clock genes, DNA‐damaging repair, oral squamous cell carcinoma, oxaliplatin

## Abstract

Developing chemotherapeutic resistance affects clinical outcomes of oxaliplatin treatment on various types of cancer. Thus, it is imperative to explore alternative therapeutic strategies to improve the efficacy of oxaliplatin. Here, it is shown that circadian regulator period 2 (PER2) can potentiate the cytotoxicity of oxaliplatin and boost cell apoptosis by inhibiting DNA adducts repair in human oral squamous cell carcinoma (OSCC) cells. The circadian timing system is closely involved in controling the activity of DNA adducts repair and gives it a 24 h rhythm. The mechanistic dissection clarifies that PER2 can periodically suppress proliferating cell nuclear antigen (*PCNA*) transcription by pulling down circadian locomotor output cycles kaput–brain and muscle arnt‐like 1 heterodimer from *PCNA* promoter in a CRY1/2‐dependent manner, which subsequently impedes oxaliplatin‐induced DNA adducts repair. Similarly, PER2 is capable of improving the efficacy of classical DNA‐damaging chemotherapeutic agents. The tumor‐bearing mouse model displays PER2 can be deployed as an oxaliplatin administration timing biomarker. In summary, it is believed that the chronochemotherapeutic strategy matching PER2 expression rhythm can efficiently improve the oxaliplatin efficacy of OSCC.

## Graphical abstract

**Circadian regulator period 2 (PER2) potentiates the cytotoxicity** of oxaliplatin depending on suppressing proliferating cell nuclear antigen (*PCNA*) transcription periodically by pulling down circadian locomotor output cycles kaput–brain and muscle arnt‐like 1 heterodimer from *PCNA* promoter in a CRY1/2‐dependent manner, which subsequently impedes oxaliplatin‐induced DNA adducts repair. Oxaliplatin administration in synergy with PER2 can efficiently improve chemotherapeutic efficacy of oral squamous cell carcinoma (OSCC).

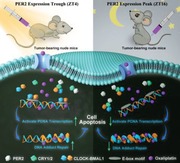

## 1 Introduction

Oral squamous cell carcinoma (OSCC) is the prevalent head and neck squamous cell carcinoma (HNSCC).^1^ Despite significant advances in clinical treatment of OSCC over the past 30 years, the prognosis of patients with OSCC remains poor.[qv: 1b,2] Thus far, chemotherapy is still the mainstream treatment for advanced OSCC whenever salvage surgery or re‐irradiation is not feasible.^2^ Oxaliplatin is a new generation platinum‐based compound that has been commonly used to treat OSCC and other types of tumors.^3^ Like other platinum drugs, oxaliplatin forms intrastrand platinum–DNA adducts and thus inhibits DNA replication and transcription.^4^ Oxaliplatin application is effective in reducing tumor size initially, inhibiting distant metastasis and prolonging patient survival.^5^ Unfortunately, the therapeutic benefits are often attenuated by the development of drug resistance.^6^ Oxaliplatin‐induced DNA damage will activate DNA repair response in cancer cells, inducing activation of adducts repair machinery.^7^ The cells can gradually improve their ability to recognize and repair DNA damage, thereby gaining the ability to resist oxaliplatin.^8^ In order to improve the therapeutic effect of oxaliplatin, combinational chemotherapy is a commonly adopted strategy.^9^ Although conventionally combinational chemotherapy can significantly improve the therapeutic efficacy, it also brings severe adverse side effects and increases the health‐care burden.^10^ Nevertheless, it is compulsory to investigate alternative oxaliplatin therapeutic strategies.

Circadian timing system (CTS) consists of a series of interlocking autoregulatory feedback loops that regulate a variety of critical biological processes rhythmically, including DNA synthesis, cell cycle, cell metabolism, apoptosis, molecular targets, drug metabolism, detoxification, etc.^11^ Genome‐wide studies have also highlighted that the majority of genes in cell cycle, DNA damage repair, cell apoptosis, and drug target are controlled by CTS, which are coordinated along the 24 h period in mammals.^12^ Our previous studies have proven that circadian clock genes are involved in the regulation of glycolysis, proliferation regulatory factors 6‐phosphofructo‐2‐kinase/fructose‐2,6‐bisphosphatase 3 (PFKFB3), and telomerase reverse transcriptase (TERT).[qv: 11c,13] Furthermore, it is found that the synergistic coupling of circadian rhythmicity of physiological activities with the application of chemotherapeutic drugs can significantly improve the treatment efficacy.[qv: 11b,14] Administration of anticancer drugs at a different circadian stage triggers notably different pharmacology and pharmacodynamics, which accounts for two‐ to tenfold changes of the drug tolerance and/or efficacy.^15^ Thus far, nearly 50 anticancer drugs have been reported to exhibit time‐dependent administration effects on their efficacy, respectively.^16^ Through selecting the appropriate medication time deliberately, maximal anticancer efficacy and minimal toxicity of these drugs can be achieved.^13^, ^14^, ^17^ In addition, DNA‐damaging repair (DDR) and DNA synthesis in cancer cells are under strict control of circadian clock genes, including circadian locomotor output cycles kaput (CLOCK), brain and muscle arnt‐like 1 (BMAL1), periods (PERs), cryptochromes (CRYs), and reverse‐erythroblastosis virus alpha (REV‐ERBα).^18^ In the light of DNA damage repair in cancer cells which is a critical influencing factor in determining the efficacy of oxaliplatin treatment,^8^ we speculated that a chronotherapeutic strategy based on the circadian features of DNA damaging repair might improve the therapeutic efficacy of oxaliplatin in OSCC. Therefore, we sought to determine the chronoinherent connection between circadian clock system and oxaliplatin efficacy and identify the modulating role of circadian clock genes in oxaliplatin sensitivity. We then explored the biological mechanism of circadian clock‐regulating oxaliplatin efficacy and established that PER2 can be regarded as a biomarker to define the appropriate therapeutic timing of oxaliplatin in OSCC.

## 2 Results

### 2.1 The Therapeutic Efficacy of Oxaliplatin is Positively Correlated with Circadian Expression of PER2 in OSCC

**Figure 1:**
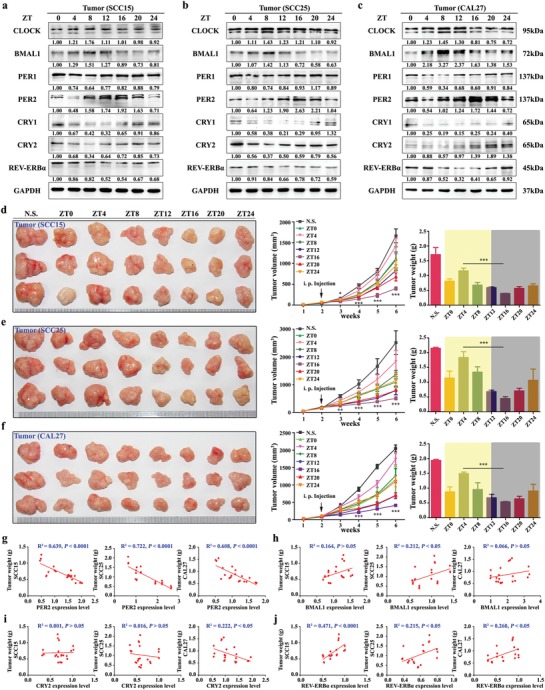
The efficacy of oxaliplatin exists in a time‐dependent manner related to PER2 expression. a–c) Western blot and densitometric quantification of circadian proteins in tumors: SCC15, SCC25, or CAL27 cells were subcutaneously injected into mice, and the tumors were obtained at indicated time points after six weeks. Samples were collected every 4 h for total 24 h. GAPDH was used for loading control. d–f) Representative images (left), tumor volume growth curves (middle), and weights (right) of tumors formed after oxaliplatin chronotherapy: SCC15, SCC25, and CAL27 cells were subcutaneously injected into mice. Two weeks after cell inoculation, mice were treated with oxaliplatin (20 mg kg^−1^, twice a week) or normal saline at indicated time points for four weeks. N.S., normal saline. *P < 0.05, **P < 0.01, and ***P < 0.001 (compared with ZT4). ANOVA and Student's *t*‐test were used. g–j) The linear correlation was analyzed by coefficient of determination between PER2, BMAL1, CRY2, and REV‐ERBα expression levels and tumor weights. Data represent the mean ± SD of three animals per group.

Circadian clock in cancer cells participates in the hallmarks of cancer, including replicative immortality, proliferative signaling maintenance, invasion and metastasis activation, cell death resistance, and energy metabolism reconfiguration.^19^ To explore the circadian characteristics of human OSCC, we first assayed the circadian rhythm of OSCC by examining the expression pattern of clock genes in xenografts from OSCC cells. Western blot analysis showed that core circadian clock proteins CLOCK, BMAL1, PER1, PER2, CRY1, CRY2, and REV‐ERBα exhibited strongly diurnal rhythms. In particular, PER2 protein level reached the peak around Zeitgeber time (ZT) 16 and the trough around ZT4 (**Figure** **1**a–c). These results were consistent with the circadian feature of PER2 expression in leukocytes from peripheral blood of tumor‐bearing mice (Figure S1a–c, Supporting Information). Likewise, synchronized OSCC cells exhibited equivalent circadian rhythms with the PER2 expression peak near circadian time 16 (CT16) and the trough near CT4 (Figure S2a–c, Supporting Information). These data indicated that there is a characteristic circadian rhythm in OSCC.

Next, we set to explore the chronomodulated feature of oxaliplatin efficacy by administering oxaliplatin according to a chronic drug‐delivery schedule. Two weeks after OSCC cell inoculation in animals, we administered a dose of 20 mg kg^−1^ oxaliplatin twice a week for a total of four weeks at the indicated time‐points. Notably, the size and weight of tumors derived from OSCC inoculation decreased significantly in the oxaliplatin treatment ZT16 group, but not in the ZT4 group (Figure 1d–f). These results were consistent with the previous report that the antitumor efficacy of oxaliplatin has diurnal fluctuation in metastatic colorectal cancer.^20^ Also, our in vitro cytotoxicity assays showed the comparable results with the prominent antitumor efficacy of oxaliplatin in the CT16 group (Figure S2d–f, Supporting Information). It is worth noting that there is a positive correlation between PER2 expression level and oxaliplatin efficacy in vivo and in vitro. In contrast, there is no consistently significant correlation between other clock genes (including BMAL1, CRY1, CRY2, REV‐ERBα) expression and oxaliplatin efficacy (Figure 1g–j and Figure S2g–i, Supporting Information). Taken together, our results suggested that OSCC cells have a chronomodulated response to oxaliplatin treatment and that this response is likely to be positively affected by the diurnal fluctuation of PER2 expression.

### 2.2 PER2 Promotes the Cytotoxicity of Oxaliplatin in OSCC

**Figure 2:**
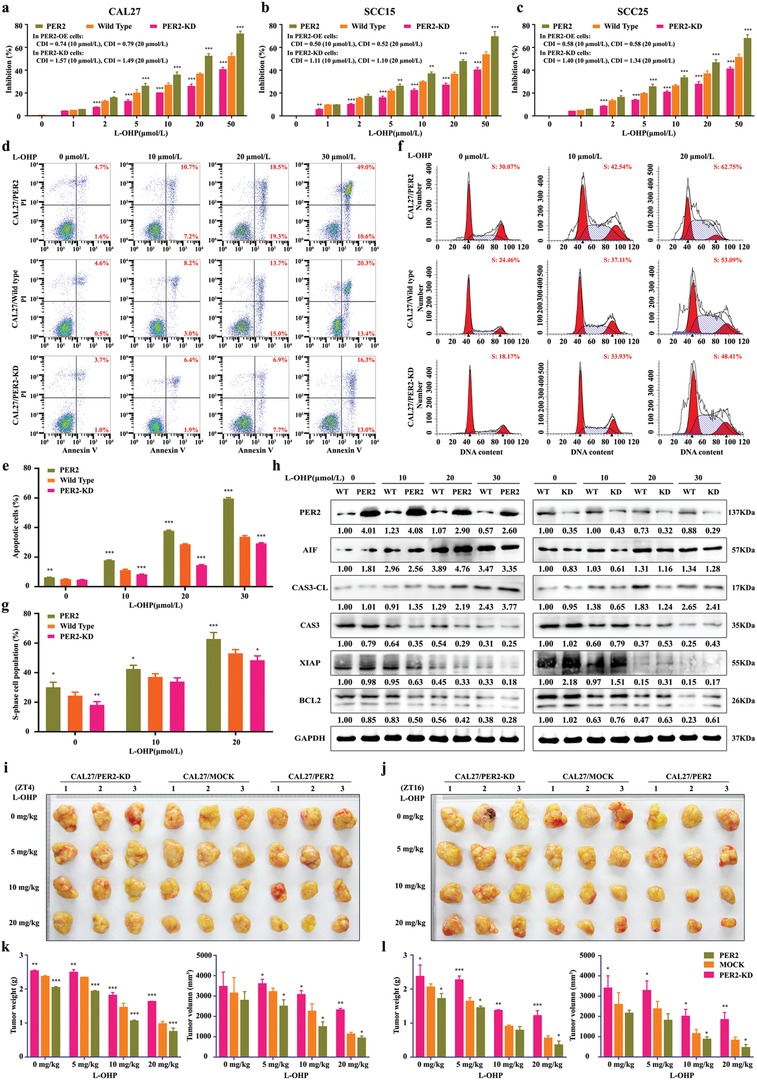
PER2 strengthens the cytotoxicity of oxaliplatin in human OSCC. a–c) Dose‐dependent growth inhibition in response to oxaliplatin (L‐OHP) treatment in PER2 overexpression or PER2‐knockdown CAL27, SCC15, and SCC25 cells (*n* = 5 independent experiments). Wild‐type cells were used as control. CDI was the coefficient of drug interaction. d,e) Cell apoptosis was evaluated by flow cytometry of CAL27/PER2 and CAL27/PER2‐knockdown (KD) cells stained with Annexin V and PI after treatment with oxaliplatin (0, 10, 20, or 30 µmol L^−1^, 48 h) (*n* = 3 independent experiments). f,g) Cell‐cycle phases were determined by flow cytometry of CAL27/PER2 and CAL27/PER2‐KD cells after treatment with oxaliplatin (0, 10, or 20 µmol L^−1^, 48 h) (*n* = 3 independent experiments). h) Western blot and densitometric quantification of the indicated proteins in CAL27/PER2 and CAL27/PER2‐KD cells treated with 0, 10, 20, or 30 µmol L^−1^ oxaliplatin (*n* = 3 independent experiments). GAPDH was used as the loading control. i,j) Representative images of xenografts formed after oxaliplatin treatment. CAL27/PER2 and CAL27/PER2‐KD cells were subcutaneously injected into mice. Mock was used as the control. g,h) Two weeks after cell inoculation, mice were treated with 0, 5, 10, or 20 mg kg^−1^ oxaliplatin (twice a week) for four weeks at ZT4 or ZT16. k,l) Tumor weights and volumes at the endpoint of mice (*n* = 3 animals per group). *P < 0.05, **P < 0.01, and ***P < 0.001 (compared with wild type or mock). ANOVA was used. Data represent the mean ± SD.

To further determine the intrinsic connection between PER2 level and the antitumor effect of oxaliplatin, we overexpressed or knocked down PER2 in OSCC cells and assayed the cytotoxicity of oxaliplatin. PER2 overexpression led to a substantial decrease in the half maximal lethal concentration value (LC_50_) of oxaliplatin, while the knockdown of PER2 reduced the cytotoxicity of oxaliplatin accordingly. In PER2‐overexpressing OSCC cells, we also found that PER2 overexpression could achieve consistently synergistic effects with oxaliplatin on cytotoxicity (coefficient of drug interaction, CDI < 1), but the cytotoxicity of oxaliplatin correspondingly was antagonized in the PER2 knockdown OSCC cells (CDI > 1) (**Figure** **2**a–c). Consistently, flow cytometry analysis showed that the overexpression of PER2 increased apoptosis of cells treated with oxaliplatin, whereas knockdown of PER2 reduced oxaliplatin‐induced OSCC apoptosis (Figure 2d,e, and Figure S3a,b,e,f, Supporting Information). Additionally, we observed the increased S‐phase cell number in PER2‐overexpressing group and the decreased S‐phase cells in a PER2 knockdown group (Figure 2f,g, and Figure S3c,d,g,h, Supporting Information). Biochemical assay indicated that the expression levels of pro‐apoptotic proteins apoptosis‐inducing factor (AIF) and Caspase 3‐cleaved (CAS3‐CL) increased in PER2‐overexpressing OSCC cells. In contrast, the levels of anti‐apoptosis proteins Caspase 3 (CAS3), X‐linked inhibitor of apoptosis (XIAP), and apoptosis regulator BCL2 were downregulated. There is an opposite trend in oxaliplatin‐treated PER2 knockdown OSCC cells, in which the pro‐apoptotic proteins level decreased (Figure 2h, and Figure S3i,j, Supporting Information).

As mentioned earlier, the size and weight of tumors derived from OSCC decreased considerably in the oxaliplatin‐treated ZT16 xenografts (Figure 1d–f). We set to assay the effect of PER2 expression on tumor weight of subcutaneous xenografts formed by oxaliplatin‐treated OSCC cells. First, we observed a significant reduction in the tumor weight of xenografts upon overexpression of PER2 and an apparent tumor weight increase in PER2 knockdown xenografts (Figure 2i–l). It should be noted that PER2 overexpression did not alter its phase. In a CAL27/PER2‐knockdown group, the PER2 circadian phenotype was lacking (data not shown). Importantly, oxaliplatin treatment at ZT16 resulted in further atrophy of the xenografts compared to the treatment at ZT4 in both the mock group and the PER2 overexpression group (Figure 2i–l). Consistently, Zeng et al. reported that BMAL1, a transcriptional activator of PER2, increased oxaliplatin sensitivity of colorectal cancer.^21^ Taken together, our results revealed that PER2 has significantly synergistic effects on the cytotoxicity with oxaliplatin and a positive correlation exists between PER2 expression pattern and oxaliplatin efficacy.

### 2.3 PER2 Enhances Oxaliplatin Sensitivity of OSCC by Inhibiting Endogenous DNA Adducts Repair

**Figure 3:**
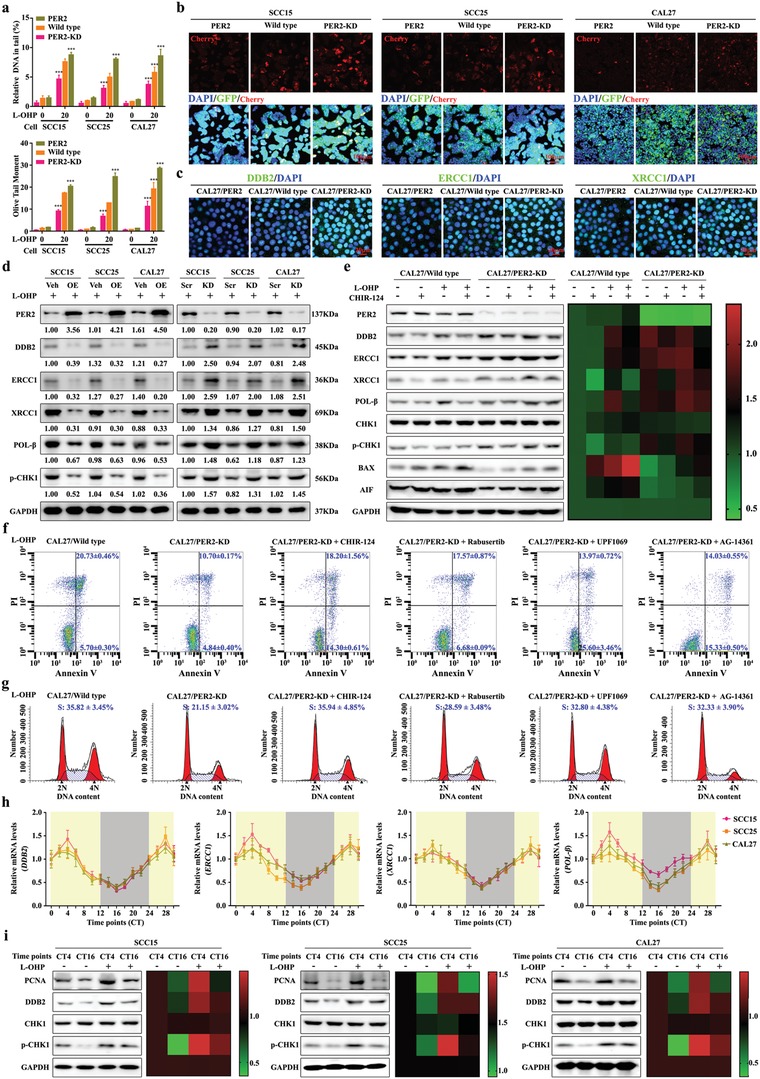
PER2 promotes the oxaliplatin sensitivity via impairing DNA adducts repair. a) Comet assay breaks. DNA strand breaks of globally oxaliplatin‐treated (30 µmol L^−1^, 48 h). Breaks were quantified as % tail DNA and olive tail moment. At least 50 cells were analyzed per sample. b) Analysis of fluorescent protein expression from oxaliplatin‐incubated vector with cherry in OSCC cells (final concentration: 50 µmol L^−1^, 12 h, at 37 °C). The vector with GFP was used as control. Scale bar, 100 µm. c) Representative confocal images of DDB2, ERCC1, and XRCC1 in PER2‐overexpressing or knockdown CAL27 cells. Scale bar, 20 µm. d) Western blot and densitometric quantification of the indicated proteins of DNA adducts repair markers in PER2‐overexpressing (left) or PER2‐knockdown (right) SCC15, SCC25, and CAL27 cells with oxaliplatin (20 µmol L^−1^) treatment. GAPDH was used as the loading control. e) Western blot (left) and densitometric quantification (right) of the indicated proteins of DNA adducts repair markers in PER2‐knockdown or control CAL27 cells after treatment with oxaliplatin (L‐OHP, 20 µmol L^−1^) with or without CHIR‐124 (0.3 nmol L^−1^). f) Apoptosis was evaluated by flow cytometry of CAL27 cells after oxaliplatin (20 µmol L^−1^) treatment with or without CHIR‐124 (0.3 nmol L^−1^), Rabusertib (7 nmol L^−1^), UPF1069 (0.3 µmol L^−1^), or AG‐14361 (4 nmol L^−1^). Cells stained with Annexin V and PI. g) Cell‐cycle phases were determined by flow cytometry of cells treated with oxaliplatin (20 µmol L^−1^). h) The circadian oscillation of *DDB2, ERCC1, XRCC1*, and *POL*‐β mRNA levels in OSCC cells at the indicated time points. i) Western blot and densitometric quantification of the indicated proteins of DNA adducts repair markers in OSCC cells after treated with or without oxaliplatin (20 µmol L^−1^) at indicated time points. GAPDH was used as the loading control. *P < 0.05, **P < 0.01, and ***P < 0.001 (compared with wild type). ANOVA was used. Data represent the mean ± SD of three independent experiments.

Oxaliplatin exerts an anticancer effect by forming platinum–DNA adducts and causing DNA damage. In the meantime, cancer cells are capable of developing drug resistance to oxaliplatin by strengthening the ability to repair DNA adducts.^22^ Importantly, compared with vehicle or scramble OSCC cells, we found that overexpression of PER2 resulted in a significant increase of DNA damage and an obvious decrease of DNA adducts repair (**Figure** **3**a,b). DNA adducts repair markers such as DNA damage‐binding protein 2 (DDB2), excision repair cross‐complementing group 1 (ERCC1), X‐ray repair cross complementing 1 (XRCC1), DNA polymerase β (POL‐β), and phosphorylated of serine/threonine‐protein kinase Chk1 (p‐CHK1) were visibly downregulated in oxaliplatin‐treated PER2‐overexpressing cells (Figure 3c,d). Conversely, the activity of DNA adducts repair and the levels of these markers were significantly elevated in PER2 knockdown OSCC cells (Figure 3a–d). It is worth noting that the apoptosis markers BAX and AIF showed a trend of change in expression level opposite to the DNA adducts repair marker (Figure 3e). The above data suggested that DNA adducts caused by oxaliplatin treatment cannot be effectively repaired due to overexpression of PER2, and eventually lead to a high level of apoptosis. In fact, after DNA adducts repair inhibitors such as CHIR‐124, Rabusertib, UPF1069, or AG‐14361 were added into oxaliplatin‐treated PER2 knockdown cells, the attenuated oxaliplatin sensitivity was significantly reversed (Figure 3e–g).

Moreover, we noticed that the expression levels of DNA adducts repair markers *DDB2, ERCC1, XRCC1*, and *POL‐β* had circadian oscillations in synchronized OSCC cells (Figure 3h). The DNA adducts repairability of oxaliplatin‐treated cells was significantly stronger than that of CT16 during CT4 (Figure 3i). Taken together, our data suggested that PER2 expression can greatly promote the oxaliplatin sensitivity of OSCC cells by inhibiting DNA adducts repair.

### 2.4 PER2 Downregulates the Expression of DNA Adduct Repair Factor Proliferating Cell Nuclear Antigen (PCNA)

**Figure 4:**
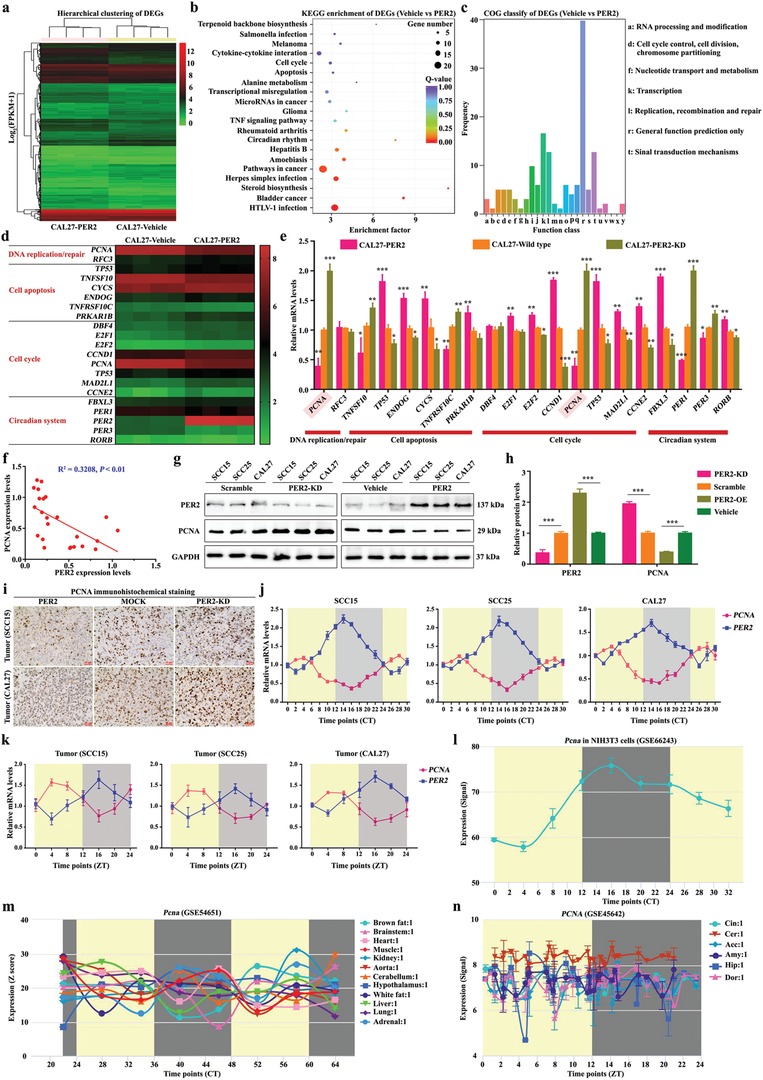
PCNA is under the strict control of the PER2‐mediated circadian clock system. a) Hierarchical clustering of differentially expressed genes (DEGs) in PER2‐overexpressing and vehicle cells (*n* = 4 per group). b,c) The KEGG enrichment and COG classify analyses of DEGs. d) The heatmap shows DEGs which were related to DNA replication and repair, cell apoptosis, cell cycle, and the circadian system. e) Confirmation of the DEGs by qRT‐PCR analysis (*n* = 3 for each bar). f) The correlation between PER2 and PCNA expression levels in human OSCC tissues (*n* = 24 samples). g,h) Western blot and densitometric quantification of PER2, PCNA, and GAPDH (as the loading control) (*n* = 3 independent experiments). i) Representative immunohistochemistry images of PCNA^+^ cells in xenografts formed by subcutaneous injection of PER2 overexpressed, PER2 knockdown, or mock SCC15 (upper)/CAL27 (lower) cells (*n* = 6 animals per group). j) The mRNA levels of *PER2* and *PCNA* in OSCC cells at indicated time‐points (*n* = 3 independent experiments). k) The mRNA levels of *PER2* and *PCNA* in OSCC xenografts at the indicated time points (*n* = 3 animals per time point). l–n) Expression pattern of rhythmic gene *Pcna* using public datasets of NIH3T3 cells (GSE66243), 12 mouse organs (GSE66243), and 6 brain regions (GSE66243). *P < 0.05, **P < 0.01, and ***P < 0.001 (compared with scramble, vehicle, or wild type), from Student's *t*‐test or ANOVA. Data represent the mean ± SD.

To gain insight into the mechanism by which PER2 modulates oxaliplatin sensitivity, we performed genome‐wide RNA‐sequencing to acquire a transcriptional profile of PER2‐overexpressing OSCC cells (**Figure** **4**a). Expression of a total of 672 genes was significantly affected in PER2‐overexpressing OSCC cells. KEGG enrichment and COG classify analyses were performed to enrich the differentially expressed genes involved in the biological processes such as cell cycle, cell division, apoptosis, DNA replication and repair, and circadian rhythm (Figure 4a–c). To further identify the potential PER2 target genes, we focused on the gene sets that are closely correlated with DDR and cell apoptosis (Figure 4d). Then, quantitative reverse transcription‐polymerase chain reaction (qRT‐PCRs) were performed to validate the expression variation. Among the genes we tested, the downregulation of *PCNA* expression was most significant (Figure 4e). The previous study indicated that PCNA plays functional roles in DNA replication and DDR.^23^ In particular, PCNA activation has a pivotal role in cellular tolerance to cisplatin‐induced damage.^24^ To better understand the correlation between PER2 and PCNA, we analyzed a panel of 24 primary OSCC tissues and found a certain degree of negative correlation between PER2 and PCNA expression (Figure 4f). The protein level of PCNA was greatly downregulated in PER2‐overexpressing OSCC cells and the formed subcutaneous xenografts in nude mice (Figure 4g–i).

To determine the functionality of PCNA in oxaliplatin sensitivity, we assayed the effects of PCNA expression changes on apoptosis and S‐phase arrest in oxaliplatin‐treated OSCC cells. Remarkably, PCNA knockdown led to a substantial decrease in oxaliplatin LC_50_ value, whereas the overexpression of PCNA‐induced cell oxaliplatin resistance (Figure S4a–c, Supporting Information). Consistently, flow cytometry analysis showed that oxaliplatin‐induced apoptosis boost and S‐phase arrest were significantly reduced in PCNA‐overexpressing cells (Figure S4d,e, Supporting Information). The levels of DNA adducts repair markers DDB2, ERCC1, XRCC1, POL‐β, and p‐CHK1 in PCNA knockdown cells declined when treated with oxaliplatin and significantly increased in PCNA‐overexpressing cells (Figure S4f,g, Supporting Information). Instead, the immunofluorescence signal of DDB2 decreased in oxaliplatin‐treated PCNA knockdown cells (Figure S4h, Supporting Information). We compared the trends in apoptosis protein BAX level and DNA adducts repair markers and found that they changed inversely (Figure S4f,g, Supporting Information). Overall, the results of the above study suggested that DNA adducts repairing process is suppressed in oxaliplatin‐treated PCNA knockdown cells, which in turn aggravates cell apoptosis. We then added CHIR‐124 to repress DNA adducts repair in oxaliplatin‐treated PCNA‐overexpressing cells and found that the attenuated oxaliplatin sensitivity caused by PCNA overexpression was reversed (Figure S4i, Supporting Information). Taken together, our finding demonstrated that PCNA can reduce oxaliplatin sensitivity caused by DNA adducts repair activation. The effect is similar to the impact of PER2 knockdown.

**Figure 5:**
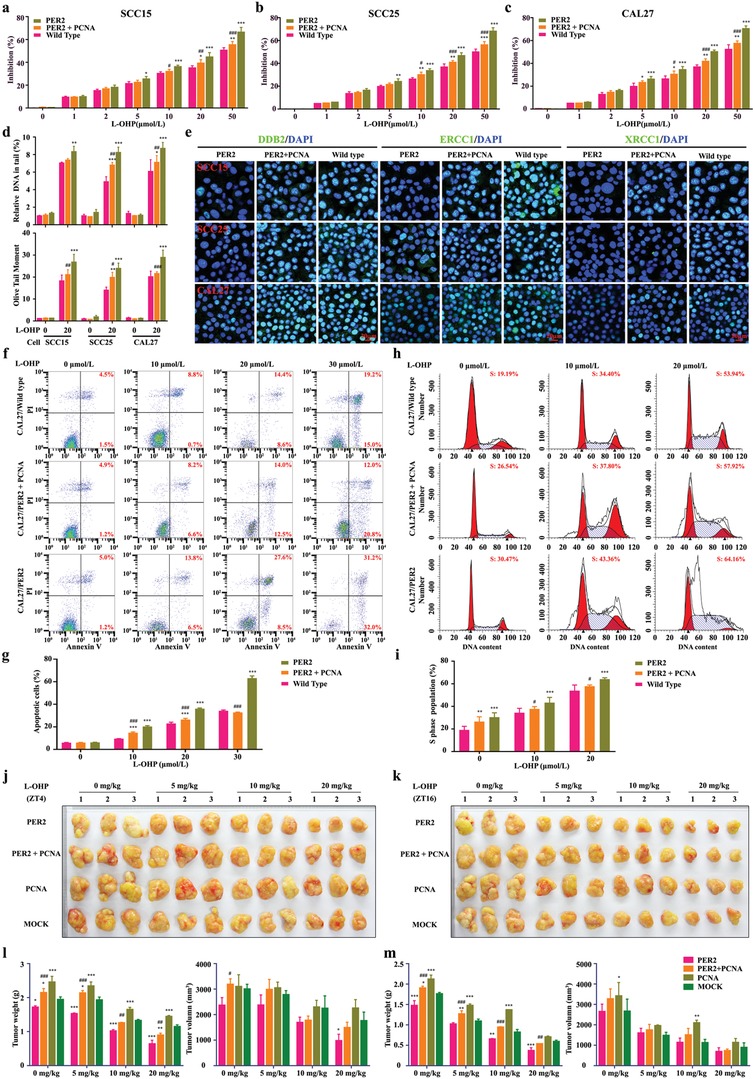
PCNA is negatively correlated with PER2 in oxaliplatin sensitivity modulation. a–c) Dose‐dependent growth inhibition in response to oxaliplatin (20 µmol L^−1^) in PER2‐overexpressing or PER2/PCNA double‐overexpressing SCC15, SCC25, or CAL27 cells (*n* = 5 independent experiments). d) Comet assay breaks. DNA strand breaks of globally oxaliplatin‐treated (30 µmol L^−1^, 48 h). Breaks were quantified as % tail DNA (upper) and olive tail moment (lower). At least 50 cells were analyzed per sample. e) Representative confocal images of DDB2, ERCC1, and XRCC1 in PER2‐overexpressing or PER2/PCNA double‐overexpressing OSCC cells (*n* = 3 independent experiments). Scale bar, 20 µm. f,g) Apoptosis was evaluated by flow cytometry of CAL27/PER2 and CAL27/PER2+PCNA cells after treatment with oxaliplatin (0, 10, 20, or 30 µmol L^−1^, 48 h), cell stained with Annexin V and PI (*n* = 3 independent experiments). h,i) Cell‐cycle phases were determined by flow cytometry of CAL27/PER2 and CAL27/PER2+PCNA cells after treatment with oxaliplatin (0, 10, or 20 µmol L^−1^, 48 h) (*n* = 3 independent experiments). j,k) Representative images of xenografts formed after oxaliplatin treatment. CAL27/PER2, CAL27/PER2+PCNA, or CAL27/PCNA cells were subcutaneously injected into mice. Two weeks after cell inoculation, mice were treated with 0, 5, 10, or 20 mg kg^−1^ oxaliplatin (twice a week) for four weeks at ZT4 (j) or ZT16 (k) (*n* = 3 animals per group). l,m) Tumor weights and volumes at the endpoint of mice. *P < 0.05, **P < 0.01, and ***P < 0.001 (compared with wild type or Mock), ^#^ *P* < 0.05, ^##^ *P* < 0.01, and ^###^ *P* < 0.001 (compared with PER2), from ANOVA. Data represent the mean ± SD.

To validate the functional association of PER2‐PCNA signaling axis with oxaliplatin sensitivity modulation, we assayed the effects of PCNA overexpression on apoptosis and S‐phase arrest in oxaliplatin‐treated PER2 overexpression cells. PCNA overexpression led to a substantial reverse in the LC_50_ value of oxaliplatin (**Figure** **5**a–c). Accordingly, PCNA level upregulation increased DNA adducts repair and decreased cell apoptosis and S‐phase arrest in PER2 overexpression cells (Figure 5a–i and Figure S5a–h, Supporting Information). Compared with the PER2 overexpression group, we observed an increased phenotype in the tumor weight of subcutaneous xenografts formed by oxaliplatin‐treated OSCC cells overexpressing both PER2 and PCNA (Figure 5j–m, Supporting Information). These observations suggested that PCNA inactivation caused by PER2 overexpression has a vital role in oxaliplatin sensitivity improvement.

### 2.5 PER2 Dissociates CLOCK‐BMAL1 Heterodimer from *PCNA* Promoter to Repress *PCNA* Transcription

PER2 often acts as a transcription co‐repressor to restrain the transcription of clock‐controlled genes.^25^ To gain insight into the mechanism by which PER2 regulates PCNA expression, we tested whether *PCNA* is a conventional clock‐controlled gene. As expected, the expression of *PCNA* had circadian oscillations in synchronized OSCC cells, and these cells formed subcutaneous xenografts (Figure 4j,k). *PCNA* mRNA level reached its trough around CT16 in vitro and ZT16 in vivo, which is opposite to the phase of PER2 protein fluctuation (Figure 1a–c, Figure S2a–c, Supporting Information, and Figure 4j,k). Likewise, a public dataset (http://cirgrbd.biols.ac.cn/) showed that *PCNA* or *Pcna* mRNA has circadian oscillations in diverse tissues and cells (Figure 4l–n). In addition, once PER2 was knocked down, *PCNA* mRNA level reached the peaks around CT8 in vitro and ZT8 in vivo, which matched well with the expression peaks of BMAL1 and CLOCK (Figure 1a–c, and Figures S2a–c and S6a,b, Supporting Information). Thus, our data demonstrated that *PCNA* is a potential clock‐controlled gene, and its circadian rhythm is somehow opposite to the phase of PER2 expression. Also, it is worth noting that the change of *PCNA* mRNA circadian phenotype caused by the loss of both BMAL1 and/or CLOCK is worthy of scrutiny (Figure S6c–f, Supporting Information).

**Figure 6:**
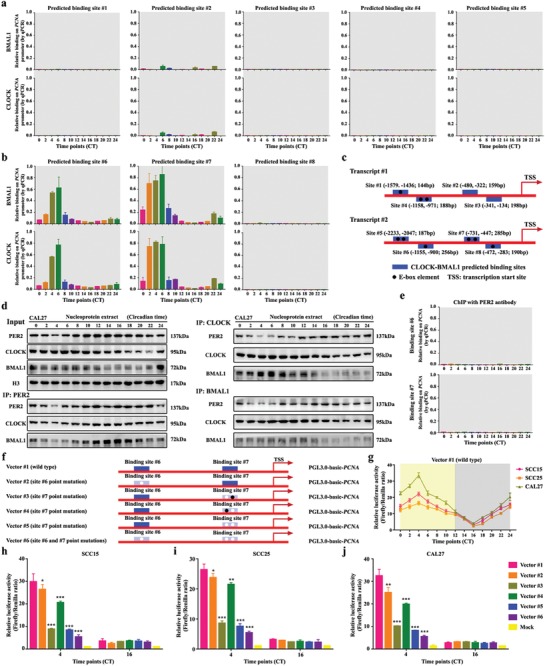
PER2 limits *PCNA* transcription by eliminating CLOCK‐BMAL1 heterodimer from *PCNA* promoter. a,b) CLOCK and BMAL1 were bound to the *PCNA* promoter at predicted binding site #6 and #7 in CAL27 cells. Chromatin immunoprecipitation assays were performed using anti‐BMAL1 or anti‐CLOCK with anti‐IgG as a negative control. c) The schematic shows the CLOCK‐BMAL1 binding sites to the *PCNA* promoter. d) Coimmunoprecipitation (Co‐IP) was performed in nucleoprotein extracts obtained across a circadian cycle with anti‐PER2, anti‐CLOCK, anti‐BMAL1 antibody, or IgG (served as a negative control) and detected by Western blot analysis with anti‐PER2, anti‐CLOCK, anti‐BMAL1, anti‐CRY1, or anti‐CRY2 antibodies. e) ChIP of PER2 on the *PCNA* promoter demonstrated that PER2 does not bind to the promoter in CAL27 cells. f) The location of the binding sites in the promoter of *PCNA*. Blue region, the location of the binding site. Filled black circle, wild‐type E‐box element. Filled white circle, point‐mutation E‐box element. g) A diurnal luciferase reporter assay was performed to measure the transcriptional activities of wild‐type *PCNA* promoter in three OSCC cells at indicated time points. h–j) A luciferase reporter assay was performed to measure the activities of wild‐type *PCNA* promoter, and its point mutations with a mutated binding site in OSCC cells at CT4 and CT16. Mock, PGL3.0‐basic. *P < 0.05, **P < 0.01, and ***P < 0.001 (compared with wild‐type vector), from ANOVA and Student's *t*‐test. Data represent the mean ± SD of three independent experiments.

To examine whether CLOCK‐BMAL1 heterodimer directly associates with the E‐box elements in the *PCNA* promoter, we used chromatin immunoprecipitation (ChIP) assay to examine OSCC cell chromatin at intervals of 2 h. The results revealed that CLOCK‐BMAL1 heterodimer could bind to E‐box elements within two regions of the *PCNA* promoter (site #6 and #7) in a highly circadian fashion. Also, the two E‐box‐containing regions are barely occupied between CT10 and CT22 (**Figure** **6**a–c). These results were consistent with the previous report that CLOCK‐BMAL1 heterodimer is associated with E‐box elements of the *PCNA* promoter of teleost.^26^

Conceivably, stripping CLOCK‐BMAL1 heterodimer from *PCNA* promoter might impede the activation of PCNA signaling. Toward this end, we deployed a co‐immunoprecipitation assay to examine the protein–protein interactions of PER2 with CLOCK or BMAL1 in the nucleus. Notably, our results showed that PER2 bound to CLOCK and BMAL1 in a highly circadian fashion. PER2 could interact more with CLOCK and BMAL1 proteins between CT10 and CT22 with high PER2 expression (Figure 6d). Indeed, anti‐CLOCK or anti‐BMAL1 antibody was capable of pulling down the PER2‐CLOCK‐BMAL1 complex. Between CT10 and CT22, PER2 can be associated with CLOCK‐BMAL1 heterodimer to the greatest extent (Figure 6d). To claim that PER2 sequestered BMAL1 and CLOCK from the *PCNA* promoter, we used ChIP assay to assess whether PER2 could bind to the *PCNA* promoter. The data showed that PER2 did not bind to the promoter, suggesting that PER2 directly interacted with CLOCK‐BMAL1 heterodimer, resulting in the immediate remove of the heterodimer from *PCNA* promoter (Figure 6e). Summarizing the results, we realized that, in the circadian clock system, the above protein–protein interactions mainly relied on the PER2 level in OSCC cells. The CLOCK‐BMAL1 heterodimer associated with the E‐box elements of *PCNA* promoter is the most abundant when PER2 is deficient, and it can be excluded from these binding sites when PER2 is abundant.

We also used a dual‐luciferase assay to assess the transcription competency of *PCNA* promoter in OSCC cells (Figure 6f–j). The results showed that the transcriptional activity of *PCNA* promoter exhibited circadian oscillations (Figure 6g), and the transcription was inhibited to some extent in the mutants with E‐box elements being mutated (Figure 6h–j). Moreover, there was little transcriptional activity of *PCNA* promoter during CT16 when PER2 was abundant (Figure 6h–j). In addition, we found that the first E‐box element in binding site #7 was the most accessible element for CLOCK‐BMAL1 heterodimer (Figure 6f–j). Together, our findings elucidated that CLOCK‐BMAL1 heterodimer activates *PCNA* transcription via associating with the E‐box elements of the promoter in the absence of PER2. Instead, abundant PER2 can inhibit *PCNA* transcription by pulling down CLOCK‐BMAL1 heterodimer from the promoter.

To further validate the repressing role of PER2 in *PCNA* transcription, we overexpressed or knocked down PER2 in OSCC cells and examined the binding state of CLOCK‐BMAL1 heterodimers. ChIP assay results showed that there was little CLOCK‐BMAL1 heterodimer coprecipitating with *PCNA* promoter in the case of elevated PER2 expression (Figure S7a, Supporting Information). Conversely, CLOCK‐BMAL1 heterodimer bound to E‐box elements within the two regions of *PCNA* promoter was added and still had a circadian fashion in the absence of PER2, in which the regions were more occupied between CT2 and CT14 when CLOCK and BMAL1 proteins were abundant (Figure S7b, Supporting Information). As CLOCK‐BMAL1 heterodimer could directly activate the transcription of *PCNA* promoter, this protein level‐dependent effect caused the circadian oscillation of *PCNA* transcription in the absence of PER2. Whereas, the circadian oscillation of *PCNA* transcription in was relatively weaker in PER2 knockdown cells (The amplitude was 1.5‐ to 2‐fold) than that in wild‐type OSCC cells (The amplitude was more than fivefold) (Figure 6b and Figure S7c–e, Supporting Information). These findings could demonstrate that PER2 is necessary to sequester BMAL1 and CLOCK from the *PCNA* promoter and suppress its transcription. Indeed, the minimum transcriptional activity of *PCNA* promoter was exhibited in PER2‐overexpressing cells and the maximum transcriptional activity of *PCNA* promoter was observed in PER2‐deficient OSCC cells (Figure S7c–e, Supporting Information). Additionally, our co‐immunoprecipitation (Co‐IP) experiments clearly showed that the interaction of PER2 and CLOCK‐BMAL1 heterodimer significantly reduced from CT2 to CT8 and increased from CT10 to CT18 in PER2 overexpressed OSCC cells which did not routinely follow with the circadian oscillation of BMAL1 protein in PER2‐overexpressing cells (Figure S7f, Supporting Information). To explain this phenomenon, we preliminarily compared the levels of *BMAL1* and *PER2*, and noticed that *PER2* was constantly at an obviously lower transcription level than that of *BMAL1*, though it was artificially overexpressed (Figure S7h, Supporting Information). Interestingly, we observed that PER2 expression still had a strongly circadian oscillation (≥3‐fold) with the peak near CT16 and the trough near CT4 in PER2‐overexpressed OSCC cells, suggesting that PER2 expression might be modulated by post‐transcriptional mechanisms which are crucial for robust circadian rhythmicity (Figure S7f, Supporting Information). These findings reminded us that PER2–CLOCK–BMAL1 interaction might mainly follow with the circadian oscillation of PER2 protein. Conversely, the interaction was absent in PER2 knockdown cells (Figure S7g, Supporting Information). Taken together, these results suggested that PER2 has a protein level‐dependent ability to exclude CLOCK‐BMAL1 heterodimer from *PCNA* promoter and periodically inhibits *PCNA* transcription in OSCC cells.

### 2.6 PER2 Eliminates CLOCK‐BMAL1 Heterodimer from *PCNA* Promoter in a CRY1/2‐Dependent Manner

**Figure 7:**
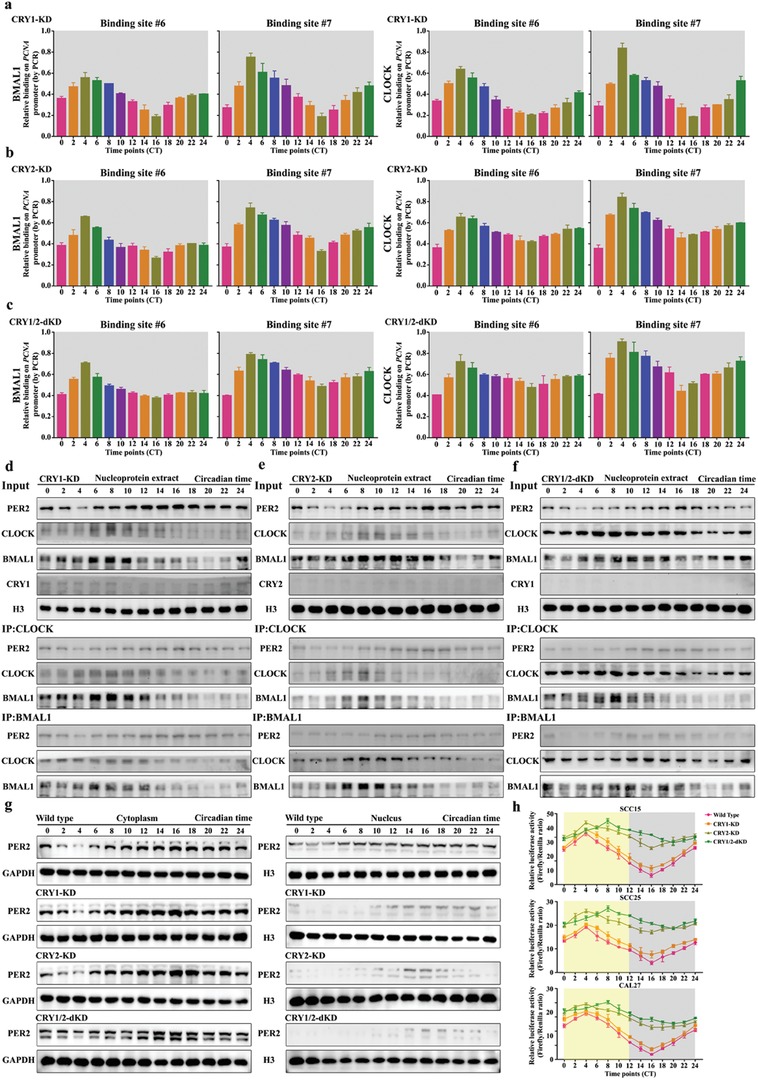
The functional process by which PER2 inhibits PCNA expression is dependent on CRY1/2. a–c) CLOCK and BMAL1 associate with *PCNA* promoter at predicted binding site #6 and #7 in CRY1 knockdown CAL27 cells, CRY2 knockdown CAL27 cells, and CRY1/2 double‐knockdown CAL27 cells at indicated time points. ChIP assay was performed using anti‐BMAL1 or anti‐CLOCK antibodies, with anti‐IgG antibody as a negative control. d–f) Coimmunoprecipitation (Co‐IP) assay was performed in nucleoprotein extracts obtained across a circadian cycle with anti‐CLOCK, anti‐BMAL1 antibody, or IgG (served as negative control) and detected by Western blot analysis with anti‐PER2, anti‐CLOCK, anti‐BMAL1, anti‐CRY1, or anti‐CRY2 antibodies in CRY1 knockdown CAL27 cells, CRY2 knockdown CAL27 cells, and CRY1/2 double‐knockdown CAL27 cells. g) Nucleocytoplasmic separation and Western blot analysis of cellular localization of PER2 in wild‐type, CRY1‐knockdown, CRY2‐knockdown, and CRY1/2 double‐knockdown CAL27 cells at indicated time points. h) A diurnal luciferase reporter assay was performed to measure the transcriptional activities of wild‐type *PCNA* promoter in human OSCC cells with CRY1‐knockdown, CRY2‐knockdown, and CRY1/2 double‐knockdown at indicated time points. Data represent the mean ± SD of three independent experiments.

CRY is known to interact with CLOCK‐BMAL1 heterodimer and regulate PER2 nucleus transferring.^27^ To investigate the role of CRYs in removing CLOCK‐BMAL1 heterodimer from *PCNA* promoter, we knocked down CRY1 and/or CRY2 in human OSCC cells and used ChIP experiments to examine the level of CLOCK‐BMAL1 heterodimers on *PCNA* promoter (**Figure** **7**a–c). When CRY1 and CRY2 were both knocked down, the two binding sites of *PCNA* promoter were largely occupied by CLOCK‐BMAL1 heterodimers over the 24 h cycle in OSCC cells, which is similar to the results in PER2‐deficient cells (Figure 7c and Figure S7b, Supporting Information). Also, the amount of PER2‐CLOCK‐BMAL1 complex significantly decreased in the absence of CRY1 or CRY2 (Figure 7d,e). PER2 could barely associate with CLOCK‐BMAL1 heterodimer in CRY1/2 double‐knockdown cells (Figure 7f). Moreover, Western blot result showed that PER2 nucleus transport was significantly inhibited in CRY1 and/or CRY2 knockdown cells (Figure 7g). Remarkably, the circadian oscillation of *PCNA* transcription was relatively weak in the CRY2 knockdown or CRY1/2 double‐knockdown cells, but not in CRY1 knockdown cells, and *PCNA* transcription reached their peak around CT8 in the absence of both CRY1 and CRY2 (Figure 7h). In addition, CRY1/2 deficiency could significantly reverse the effect of PER2 on improving the cytotoxicity of oxaliplatin (Figure S8a,b, Supporting Information). Overall, our results demonstrated that PER2 is suppressing the transcription of *PCNA* in a CRY1/2‐dependent manner.

### 2.7 PER2 Can Be Utilized as a Novel Administration Timing Biomarker to Improve the Chronochemotherapeutic Efficacy of DNA‐Damaging Agents

**Figure 8:**
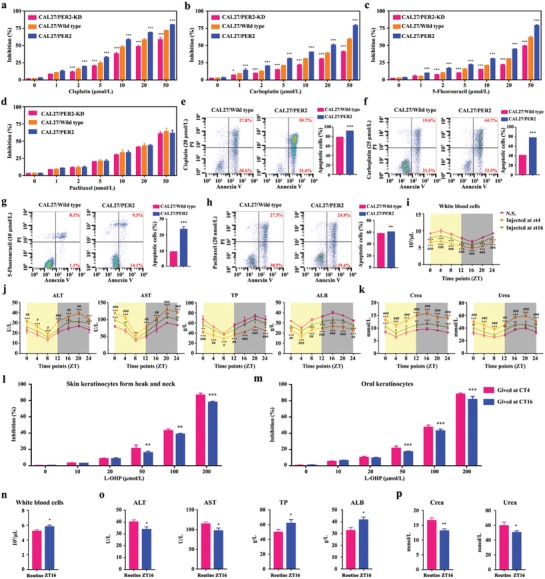
PER2 improves the efficacy and tolerance of DNA‐damaging agents. a–d) Dose‐dependent growth inhibition in response to cisplatin, carboplatin, 5‐fluorouracil, and Paclitaxel in PER2‐overexpression or PER2‐knockdown CAL27 cells (*n* = 5 independent experiments). Wild‐type cells were used as the control. e–h) Apoptosis was evaluated by flow cytometry of CAL27/wild‐type and CAL27/PER2 cells after treatment with cisplatin, carboplatin, 5‐fluorouracil, and Paclitaxel, cell stained with Annexin V and PI (*n* = 3 independent experiments). *P < 0.05, **P < 0.01, and ***P < 0.001 (compared with wild type), from ANOVA and Student's *t*‐test. i) The total number of white blood cells in mice injected with oxaliplatin (20 mg kg^−1^, twice a week) at ZT4 or ZT16 for four weeks. Normal saline was used as control. *P < 0.05, **P < 0.01, and ***P < 0.001 (compared with N.S.), ^#^ *P* < 0.05, ^##^ *P* < 0.01, and ^###^ *P* < 0.001 (compared with ZT16). j,k) Serum alanine transaminase (ALT), aspartate transaminase (AST), total protein (TP), albumin (ALB), creatinine (crea), and urea levels were measured every 4 h over a circadian period after injecting four‐week oxaliplatin at ZT4 or ZT16. l,m) Dose‐dependent growth inhibition in response to oxaliplatin (L‐OHP) treatment in normal epithelial cells from the OSCC adjacent tissues (*n* = 5 independent experiments). **P < 0.01 and ***P < 0.001 (compared with ZT4). n–p) White blood cells and serum ALT, AST, TP, ALB, crea, and urea levels were measured in mice with injecting four‐week oxaliplatin at ZT16 or random time (routine). *P < 0.05 and **P < 0.01 (compared with routine), from Student's *t*‐tests. Data represent the mean ± SD of three independent experiments.

DDR is a common mechanism used by cells to resist DNA‐damaging agents. Here, we found that PER2 can repress DDR, suggesting that PER2 has the synergistic capacity in improving the efficacy of conventional DNA‐damaging drugs. As expected, PER2 overexpression led to a substantial decrease of the half maximal lethal concentration value (LC_50_) of cisplatin, carboplatin, and 5‐fluorouracil, while the knockdown of PER2 significantly reduced the cytotoxicity of these drugs (**Figure** **8**a–c). Compared with control cells, we also noted that there was no significant difference in the LC_50_ of paclitaxel in OSCC cells with PER2 overexpression or knockdown (Figure 8d). Accordingly, the overexpression of PER2 led to increased apoptosis in cells treated with DNA‐damaging drugs including cisplatin, carboplatin, and 5‐fluorouracil, but drugs that do not target DNA, such as paclitaxel, failed to augment apoptosis (Figure 8e–h). Taken together, our results revealed that PER2 has significantly synergistic effects on the cytotoxicity with DNA‐damaging drugs and a positive correlation exists between PER2 expression pattern and the efficacy.

Meanwhile, the tolerability of oxaliplatin injected at the peak and the trough of PER2 was compared in normal tissues, including peripheral blood, liver, kidney, and the surrounding epithelium. Consistently, we observed less leucopenia and slighter hepatoxicity and nephrotoxicity in mice when oxaliplatin was injected at ZT16, as compared with that at ZT4 (Figure 8i–k). Expectedly, the effective concentration of oxaliplatin to kill OSCC cells could not result in a significantly lethal cytotoxicity to normal epithelial cells from the OSCC adjacent tissues, and LC_50_ of oxaliplatin in normal epithelial cells was substantially increased when oxaliplatin was given at CT16, compared with that at CT4 (Figures 2a–c and 8l,m). Remarkably, chronomodulated chemotherapy of oxaliplatin injected at the peak of PER2 was safer in the treatment of OSCC, compared with conventional delivery (Figure 8n–p). Meanwhile, the efficacy of personalized chronochemotherapeutic strategy matching the circadian pace of PER2 was optimal (Figure 1a–j). Taken together, our data indicated that PER2 is an effective modulator of DNA‐damaging agents, whose efficacy can be greatly boosted with timely administration at the peak of PER2 expression. Since the circadian feature of PER2 expression in peripheral blood of tumor‐bearing mice is consistent with that in OSCC tissue (Figure S1a–c, Supporting Information), it is feasible to utilize PER2 in peripheral blood as a biomarker to guide chronomodulated drug delivery of DNA‐damaging agents.

## 3 Discussion

To explore the new strategy for improving oxaliplatin sensitivity of human OSCC, we performed a series of studies and found that transcription cofactor PER2 could potentiate the cytotoxicity of oxaliplatin via deregulating PCNA expression, thereby suppressing the activity of DNA adducts repair. Actually, the administration of oxaliplatin at CT16 showed more than twofold effect on apoptosis than that at CT4 in cultured OSCC cells. The overall efficacy on the tumor growth by oxaliplatin injected at ZT16 was the accumulation of the effect of eight doses. Our mechanistic dissection clarified that PER2 could periodically repress *PCNA* transcription by excluding CLOCK‐BMAL1 heterodimer from the promoter in a CRY1/2‐dependent manner, thus impeding oxaliplatin‐induced DNA adducts repair. Based on the above results, our research proposes a new chronochemotherapeutic strategy to regard peripheral blood PER2 as a biomarker for timely oxaliplatin application with improved chemotherapy efficacy in OSCC.

Oxaliplatin is the currently used first‐line chemotherapy drug, which is mainly used for the treatment of patients with advanced OSCC cancer and other solid tumors.[qv: 3b,28] However, acquired resistance is the main hurdle for oxaliplatin application.^29^ Previous research pointed out that oxaliplatin‐induced DNA damage is primarily fixed by nucleotide excision repair (NER) system in cancer cells.^22^ One of the most important NER mediators, ERCC1, has been proposed to be a predictor of disfavored response in oxaliplatin‐treated patients.^30^ The siRNA‐mediated gene silencing has been used to improve oxaliplatin sensitive by inhibiting the molecular process of DNA adducts repair in oxaliplatin‐treated cells.^31^ Currently, combinational therapy with additional chemotherapeutic drugs, such as 5‐fluoropyrimidine, is the mainstream treatment strategy for improving patients survival rate. Nevertheless, combinational chemotherapy often causes a series of adverse reactions and leads to an increased health‐care burden.^10^ Chronochemotherapeutic strategy refers to selecting the appropriate medication time and has been widely considered as an effective way to maximize the benefits and minimize toxicities simultaneously.^14^ Thus, it is appealing to improve oxaliplatin sensitivity through timely administration of oxaliplatin. However, the indiscriminate chronotherapy infusion scheme for all patients could significantly produce patient‐to‐patient variability in treatment outcomes. For example, in the case of metastatic colorectal cancer, the rigid three‐drug chronotherapy schedule (chronomodulated infusion of fluorouracil, leucovorin, and oxaliplatin for 4 d, chronoFLO4) increased the survival rate of male patients but reduced that of female patients.^32^ Circadian rhythms in body are affected by genetic background and environment. Thus, there is significant interpatient variability in the schedule of timely medication.^32^, ^33^ The personalized administration schedule of chronotherapy, a dedicated treatment, is promising to increase patient response to oxaliplatin application. Toward this end, we explored the possibility of using circadian clock genes as biomarkers to optimize the efficacy of oxaliplatin and identified the positive correlation between PER2 expression and oxaliplatin sensitivity of OSCC cells. Although PER2 expression was lower in OSCC than those in normal head and neck tissues, its 24 h oscillation was three‐ to fivefold in OSCC tissue.[qv: 11c,13] The expression peak of PER2 is the appropriate administration time‐point for personalized oxaliplatin treatment. Therefore, the circadian factor PER2 can be deployed as a novel biomarker for the chronomodulated drug‐delivery schedule of oxaliplatin in OSCC.

Recently, the role of circadian clock in DDR has been attracting the attention of the field due to its implication in cancer therapy.^34^ Nonetheless, the molecular mechanism of circadian clock‐regulating DDR remains vague.^35^ Our results indicated that DDR has circadian oscillation in synchronized OSCC cells. The repair process initiates during PER2 expression trough (ZT4) and slows down during PER2 expression peak (ZT16), suggesting that PER2 can influence DDR negatively. Consistently, it has been reported that Per2‐deficient mice maintain vigorous DDR and the lengthy cancer‐free lifespan.^36^ Previous studies showed that the oscillation of DDR could be ascribed to the circadian rhythm of XPA (DNA damage recognition and repair factor) expression in antiphase with CRY1 or in normal phase with BMAL1.[qv: 18b,37] Conversely, in OSCC cells, we observed that the rhythm of DNA adducts repair markers (*DDB2, ERCC1, XRCC1*, and *POL*‐β) is in antiphase with PER2. These discrepancies could be explained by the differentiated significance of different circadian genes in various cell types, highlighting the complex involvement of circadian clock in DDR.^38^

The negative regulation of PCNA by PER2 is indispensable during oxaliplatin sensitivity modulation. PCNA can impact on a myriad of cellular processes, such as promoting DNA synthesis and DDR.^39^ Therefore, PCNA downregulation is beneficial to DNA‐damaging chemotherapy.^40^ In the process of NER, PCNA can interact with the scaffold protein XPA, thus activating the endonuclease ERCC4 and recruiting polymerase δ to the single‐stranded gap.[qv: 39b] In the current study, we showed that PER2 eliminates CLOCK‐BMAL1 heterodimer from *PCNA* promoter and curbs *PCNA* transcription in a CRY1/2‐dependent manner. In the mammal, clock protein CLOCK, BMAL1, CRY, PER, and their paralogs can form an autoregulatory feedback loop.^41^ A CLOCK‐BMAL1 heterodimer mediates the positive transcription arm of the loop via binding to E‐box motif of those clock‐controlled genes to motivate transcription.^42^ PERs and CRYs can also form a heterodimer and suppress their own expression via blocking CLOCK‐BMAL1‐mediated transcription activation.^43^ Moreover, our experiments demonstrated that PER2 restricts *PCNA* transcription periodically and causes the circadian fluctuation of oxaliplatin‐induced DDR in OSCC cells.

The circadian feature of DDR implies that the efficacy of DNA‐damaging agents can be enhanced by chronochemotherapeutic strategy. Indeed, improved outcomes of circadian‐based treatments have been demonstrated in randomized clinical trials.^44^ However, it should be noted that interpatient difference in circadian features could result in significant variability in chronotherapy response. Therefore, personalized chronochemotherapeutic strategy by the circadian pace of PER2 of an individual patient is expected to improve oxaliplatin efficacy in OSCC. This strategy could also be applied to the clinic administration of other DNA‐damaging drugs.

## 4 Conclusion

Our research proposes a new chronochemotherapeutic strategy to regard peripheral blood PER2 as a biomarker for timely oxaliplatin or other DNA‐damaging drugs application with improved chemotherapy efficacy in OSCC.

## 5 Experimental Section

*Mice*: A cohort of 780 male BALB/c nude mice (4‐week‐old) was purchased. All mice were placed under 12 h light/12 h dark schedule conditions with the light on from 8 a.m. (zeitgeber time 0: ZT0) to 8 p.m. (ZT12) and fed with water and antibiotic‐free food ad libitum. All animal experiments were carried out with the consent of the Institutional Animal Care and Use Committee. Six in vivo experiments were designed. In experiment 1, SCC15, SCC25, or CAL27 cell suspension (1 × 10^7^ cells in 300 µL of saline) was subcutaneously injected into mice, and the tumors were obtained after six weeks at indicated time points (ZT0, 4, 8, 12, 16, 20, and 24) to compare the expressions of indicated clock proteins (*n* = 5 animals per time point per cell line). In experiment 2, SCC15, SCC25, or CAL27 cells were subcutaneously injected into mice. Two weeks after cell inoculation, mice were treated with oxaliplatin (20 mg kg^−1^, twice a week) or normal saline at indicated time points for four weeks. Then the mice were sacrificed to compare tumor weight and volume (*n* = 5 animals per time point per cell line). In experiment 3, the mice were randomly divided into CAL27/mock, CAL27/PER2 (PER2 overexpression), and CAL27/PER2‐KD (PER2 knockdown) groups. Two weeks after cell inoculation, mice were treated with oxaliplatin (0, 5, 10, or 20 mg kg^−1^, twice a week) at ZT4 or ZT16 for four weeks. Then the mice were sacrificed to compare tumor weight and volume (*n* = 5 animals per treatment per group). In experiment 4, the mice were randomly divided into CAL27/mock, CAL27/PER2 (PER2 overexpression), CAL27/PER2+PCNA (PER2 and PCNA double‐overexpression), or CAL27/PCNA (PCNA overexpression) groups. Two weeks after cell inoculation, mice were treated with 0, 5, 10, or 20 mg kg^−1^ oxaliplatin (twice a week) for four weeks at ZT4 or ZT16. Then the mice were sacrificed to compare tumor weight and volume (*n* = 5 animals per treatment per group). In experiment 5, CAL27 cells were subcutaneously injected into mice. Two weeks after cell inoculation, mice were treated with oxaliplatin (20 mg kg^−1^, twice a week) or normal saline at ZT4 or ZT16 for four weeks. Then the mice were sacrificed to harvest blood sample every 4 h over a period of 24 h. White blood cells, hepatic and renal functions were detected using an Automatic Blood Cell Analyzer (Rayto, China) (*n* = 5 animals per time point per group). In experiment 6, CAL27 cells were subcutaneously injected into mice. Two weeks after cell inoculation, mice were treated with oxaliplatin (20 mg kg^−1^, twice a week) at ZT16 or random time for four weeks. Then the mice were sacrificed to detect white blood cells, hepatic and renal functions (*n* = 5 animals per group). The width and length of the tumors were obtained with digital calipers, and the volume was calculated (*V* (mm^3^) = 0.5326 × length × width^2^).

*Cell Lines*: Human OSCC cell lines SCC15, SCC25, and CAL27 were obtained from American Type Culture Collection (ATCC) and authenticated by short tandem‐repeat DNA fingerprinting. SCC15 and SCC25 cells were cultured in DMEM/Ham's F‐12 medium (Hyclone) with 10% fetal bovine serum (FBS) (Gibco), 1.2 g L^−1^ sodium bicarbonate (Sigma), 2.5 mmol L^−1^ l‐glutamine (Sigma), 15 mmol L^−1^ HEPES (Sigma), and 0.5 mmol L^−1^ sodium pyruvate (Sigma) supplemented with 400 ng mL^−1^ hydrocortisone (Sigma). CAL27 cells were cultured in DMEM (Hyclone) with 10% FBS. All cells were incubated at 37 °C in 5% CO_2_.

*Culture and Identification of Keratinocytes*: Human head and neck keratinocytes (SKCs) and oral keratinocytes (OKCs) were isolated and cultured as previously described.^13^ Both of them were authenticated by immunofluorescence assay with the antibodies of anti‐cytokeratin 19 (Proteintech, China) and anti‐vimentin (Proteintech, China). This study was approved by the Institutional Research Ethics Committee of Tongji Medical College (Wuhan, China) and that the informed consent of all participating subjects was obtained.

*Circadian Rhythm Induction*: The medium was removed from 100% confluent cultures of cells in 6‐well or 12‐well plates and replaced with fresh medium containing 1 µmol L^−1^ dexamethasone (Sigma). The cells were exposed to the inductive agents for 2 h, followed by replacing with fresh complete medium (set this timing as circadian time 0, CT0). The cells did not receive any further medium changes from this time point onward until the time of harvest. Individual cells were harvested for total RNA and protein at CT0, 2, 4, 6, 8, 10, 12, 14, 16, 18, 20, 22, and 24.

*Viral Infection*: Viral packaging was purchased. For viral inoculation, SCC15, SCC25, or CAL27 cells were incubated with the viral supernatants (MOI = 10) in complete medium for 24 h. Medium was switched to standard culture media 24 h post infection. The vector information is listed in the Key Resources Table.

*Quantitative Real‐Time PCR Analysis*: RNA extraction was isolated from cells or tissues with TRIZOL (TAKARA) according to the manufacturer's protocol. RNA samples were reverse‐transcribed using the HiScript II Q RT SuperMix for qPCR kit (Vazyme). Quantitative real‐time PCR was performed using SYBR Green PCR Master Mix (Applied Biosystems). Relative expression level was analyzed by the 2^−△△Ct^ method, normalized to expression of GAPDH, and presented as mean ± SD of replicates. The sequences of the primers used for qRT‐PCR are listed in Table S1 (Supporting Information).

*Western Blot Analysis*: Cells were lysed in RIPA buffer with 1 mmol L^−1^ phenylmethylsulfonyl fluoride (PMSF), normalized by BCA protein kit, and degenerated by sodium dodecyl sulfate polyacrylamide gel electrophoresis (SDS‐PAGE) loading buffer. Total protein extracts were resolved on 10% SDS‐PAGE, and transferred to polyvinylidene fluoride (PVDF) membranes. Membranes were blocked with 5% skim milk for 1 h at room temperature, and then incubated with primary antibodies (Table S2, Supporting Information) at 4 °C overnight. ECL enhanced chemiluminescence substrate kit (Millipore) was used for imaging and quantitation after incubated with secondary antibodies by the Image J software.

*Immunofluorescence*: Cells seeded in 24‐well plates were fixed with 4% paraformaldehyde for 15 min. After washed with PBS for three times, fixed cells were then permeabilized with PBS including 0.1% Triton X‐100 for 10 min at room temperature. Being washed three times again, cells were blocked with 5% bovine serum albumin (BSA, Sigma) in PBST for 1 h at room temperature and incubated with DDB2, ERCC1, and XRCC1 antibodies (Table S2, Supporting Information) overnight at 4 °C later. After being washed with PBS, they were incubated with Alexa Flour 488‐conjugated goat anti‐rabbit IgG (Proteintech, China) for 1 h. Nuclei were counterstained with DAPI (Sigma), and images were captured using a laser scanning confocal microscope (Nikon A1‐Si, Japan).

*Immunohistochemistry*: The xenografts were isolated, fixed with 10% buffered formalin overnight, dehydrated with 70% ethanol, embedded in paraffin, and sectioned in sequence. Then, sections were incubated with primary antibodies specific for PCNA (Abcam, 1:1000) at 4 °C overnight, following incubated with biotinylated secondary antibodies (Abcam, 1:500). Stained sections were scanned by a ScanScope XT (Aperio Technologies, Singapore).

*Cell Cytotoxicity Assays*: Cell counting kit (CCK‐8, Beyotime Institute of Biotechnology) was used to detect cellular proliferation and cytotoxicity by manufacturer's instructions. The cells were plated at a density of 2000 cells per well in 96‐well plates and incubated for 48 h. For cytotoxicity assay, the cells were incubated with a different concentration of oxaliplatin (Selleck Chemicals, China). The absorbance was studied at 450 nm wavelength. The CDI was used to analyze the interaction of PER2 and oxaliplatin.^45^ CDI was calculated as follows: CDI = *AB*/(*A* × *B*). CDI < 1 indicated a synergistic effect, CDI = 1 indicated additivity, and CDI > 1 indicated antagonism. *AB* was the ratio of the combination group of PER2 overexpression/knockdown and oxaliplatin to the control group; both *A* and *B* were the ratios of each of the single‐drug groups to the control group.

*Comet Assay*: The presence of DNA breaks was analyzed using the Comet Assay kit from Cell Biolabs according to the manufacturer's instructions. In brief, cells were combined with Comet Agarose and spread onto three‐well Comet Assay slides, solidified at 4 °C. Slides were electrophoresed in chilled Tris‐borate‐EDTA (TBE) buffer, and then fixed in 70% ethanol. Once dried, DNA was labeled with Vista Green DNA Dye. Images were captured with an inverted microscope (Olympus) and analyzed using Image J software, at least 50 cells were analyzed per sample.

*DNA‐Damaging Repair Assay in Living Cells*: The assay was conducted as previously described.^46^ Vector with Cherry was incubated with oxaliplatin (final concentration: 50 µmol L^−1^) at 37 °C for 12 h and were purified with MicroSpin G‐25 Columns (GE Healthcare). Cells transfected with GFP vector were cultured on 24‐well plates until 30–50% confluent. The cherry vectors were then transfected. The images were captured using a laser scanning confocal microscope (Nikon, Japan).

*Flow Cytometry*: The cell lines were harvested after 0, 10, 20, or 30 µmol L^−1^ oxaliplatin treatment for 48 h. For cell cycle analysis, cells were fixed with ice‐cold 70% ethanol in 4 °C overnight. Cells were washed with PBS and resuspended in 200 µL PI/RNase Staining Buffer (BD Biosciences) for 30 min. About 10^4^ cells per sample were analyzed through flow cytometry (BD Biosciences). For apoptosis analysis, cells were washed with PBS and resuspended in 200 µL binding buffer, then incubated with 5 µL of Annexin V‐FITC for 15 min and 10 µL of PI (Thermo Fisher Scientific) for 5 min, finally subjected to flow cytometry analysis.

*Chromatin Immunoprecipitation*: ChIP assays were performed using Chromatin Immunoprecipitation Kit (Millipore) according to the procedures provided by the manufacturer. Chromatin solutions were precipitated using normal mouse IgG as negative control, anti‐BMAL1 (Abcam, 1:100), anti‐CLOCK (Abcam, 1:100), or anti‐PER2 (Abcam, 1:100) (Table S2, Supporting Information) at 4 °C overnight. Precipitates were analyzed by qPCR, and the primers used for detecting CLOCK‐BMAL1 binding to *PCNA* promoter region are listed in Table S1 (Supporting Information).

*Luciferase Reporter Assay*: OSCC cells were cotransfected with firefly and pRL‐SV40 renilla luciferase reporter vectors (Promega) using lipofectamine 3000 (Thermo Fisher Scientific). Cells extracts were harvested post 48 h transfection. Firefly and renilla luciferase was measured by the dual luciferase reporter assay system (Promega). For *PCNA* promoter activity, the luciferase signal was standardized according to the firefly/renilla ratio.

*Co‐Immunoprecipitation*: Nuclear proteins were extracted by nuclear and cytoplasmtic extraction reagents (Thermo Fisher Scientific), according to manufacturer's protocol. Magnetic beads were used to eliminate nonspecific bonding. About 10% supernatants were stored as “Input,” the remaining supernatants were divided into equal parts. Three parts were incubated with magnetic beads preloaded with specific primary antibody, respectively: anti‐PER2 antibody (Abcam, 1:200), anti‐CRY1 antibody (Abcam, 1:100), anti‐CRY2 antibody (Abcam, 1:100), anti‐CLOCK antibody (Abcam, 1:200), and anti‐BMAL1 antibody (Abcam, 1:200) (Table S2, Supporting Information), and the last part was incubated with IgG antibody as negative control at 4 °C for 4 h. After immunoprecipitation, beads were washed and resuspended in SDS loading buffer. The bound proteins were dissociated with magnetic beads via boiling and centrifugation. Precipitates were analyzed by Western blotting.

*RNA‐Sequencing and Analysis*: Total samples were prepared for RNA sequencing from PER2 overexpressing and vehicle CAL27 cells (four samples each group). Then the complementary DNA (cDNA) sequencing library of each RNA sample was prepared using TruSeq Stranded mRNA‐Seq Library Preparation Kit (Illumina). All RNA samples from biological replicates were prepared and subsequently analyzed using the Agilent 2100 TapeStation.

*Statistical Analysis*: Statistical significances were assessed using Student's *t*‐test or ANOVA. Results were presented as mean ± standard deviation (SD). *P* value < 0.05 was considered as statistically significant and all statistical analyses were performed with GraphPad Prism 7.0 software.

## Conflict of Interest

The authors declare no conflict of interest.

## Supporting information

Supplementary
