# Supplementary material for: Periodic Oxaliplatin Administration in Synergy with PER2‐Mediated PCNA Transcription Repression Promotes Chronochemotherapeutic Efficacy of OSCC
Source: Adv Sci (Weinh). 2019 Sep 8;6(21):1900667. doi: 10.1002/advs.201900667 (PMC6839751; doi:10.1002/advs.201900667)
Supplement: Supplementary file 1 — Supplementary [file ADVS-6-1900667-s001.pdf]

Copyright WILEY-VCH Verlag GmbH & Co. KGaA, 69469 Weinheim, Germany, 2019.

ADVANCED SCIENCE Open Access

Supporting Information

for *Adv. Sci.,* DOI: 10.1002/advs.201900667

Periodic Oxaliplatin Administration in Synergy with PER2-Mediated *PCNA* Transcription Repression Promotes Chronochemotherapeutic Efficacy of OSCC

*Qingming Tang, Mengru Xie, Shaoling Yu, Xin Zhou, Yanling Xie, Guangjin Chen, Fengyuan Guo, and Lili Chen**

Copyright WILEY-VCH Verlag GmbH & Co. KGaA, 69469 Weinheim, Germany, 2018.

Supporting Information

Title:

Periodic oxaliplatin administration in synergy with PER2-mediated PCNA transcription repression promotes chronochemotherapeutic efficacy of OSCC

*Qingming Tang, Mengru Xie, Shaoling Yu, Xin Zhou, Yanling Xie, Guangjin Chen, Fengyuan Guo, and Lili Chen**

Dr. Q. Tang, M. Xie, S. Yu, Dr. X. Zhou, Y. Xie, G. Chen, F. Guo, Prof. L. Chen

Department of Stomatology, Union Hospital, Tongji Medical College, Huazhong University of Science and Technology, Wuhan 430022, China

Email: lily-c1030@163.com

Directory listing

Supplementary Figures and Figure Legends

Figure S1. The circadian pattern of PER2 in xenografts is consistent with its expression oscillation in peripheral blood

Figure S2. Oxaliplatin chronomodulated efficacy is closely correlated with the diurnal fluctuation of PER2 expression *in vitro*

Figure S3. PER2 increases oxaliplatin sensitivity of human OSCC cells

Figure S4. PCNA enhances DNA adducts repair and impairs oxaliplatin sensitivity in human OSCC cells

Figure S5. PCNA modulates the functionality of PER2 in oxaliplatin sensitivity

Figure S6. Circadian proteins regulate the diurnal fluctuation of *PCNA* transcription

Figure S7. Overexpressed PER2 suppresses *PCNA* transcription through removing CLOCK-BMAL1 heterodimers from its promoter extensively

Figure S8. CRYs correlate with PER2 in oxaliplatin sensitivity modulation

Supplementary Tables

Table S1. Primer sequences used in qRT-PCR and ChIP assays

Table S2. Antibodies used in this study

Supplementary Figures and Figure Legends

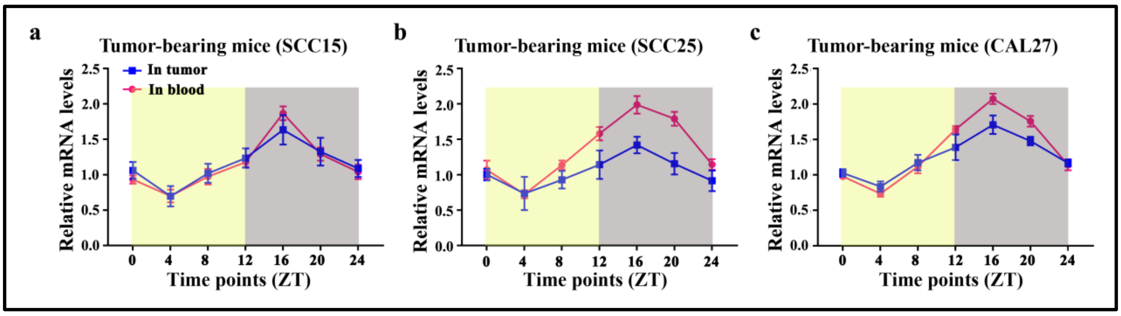

Figure S1. The circadian pattern of PER2 in xenografts is consistent with its expression oscillation in peripheral blood

a) A circadian feature of PER2 in the xenografts and blood of tumor-bearing mice (SCC15).

b) A circadian feature of PER2 in the xenografts and blood of tumor-bearing mice (SCC25).

c) A circadian feature of PER2 in the xenografts and blood of tumor-bearing mice (CAL27).

Data represent the mean ± SD of 3 animals per group.

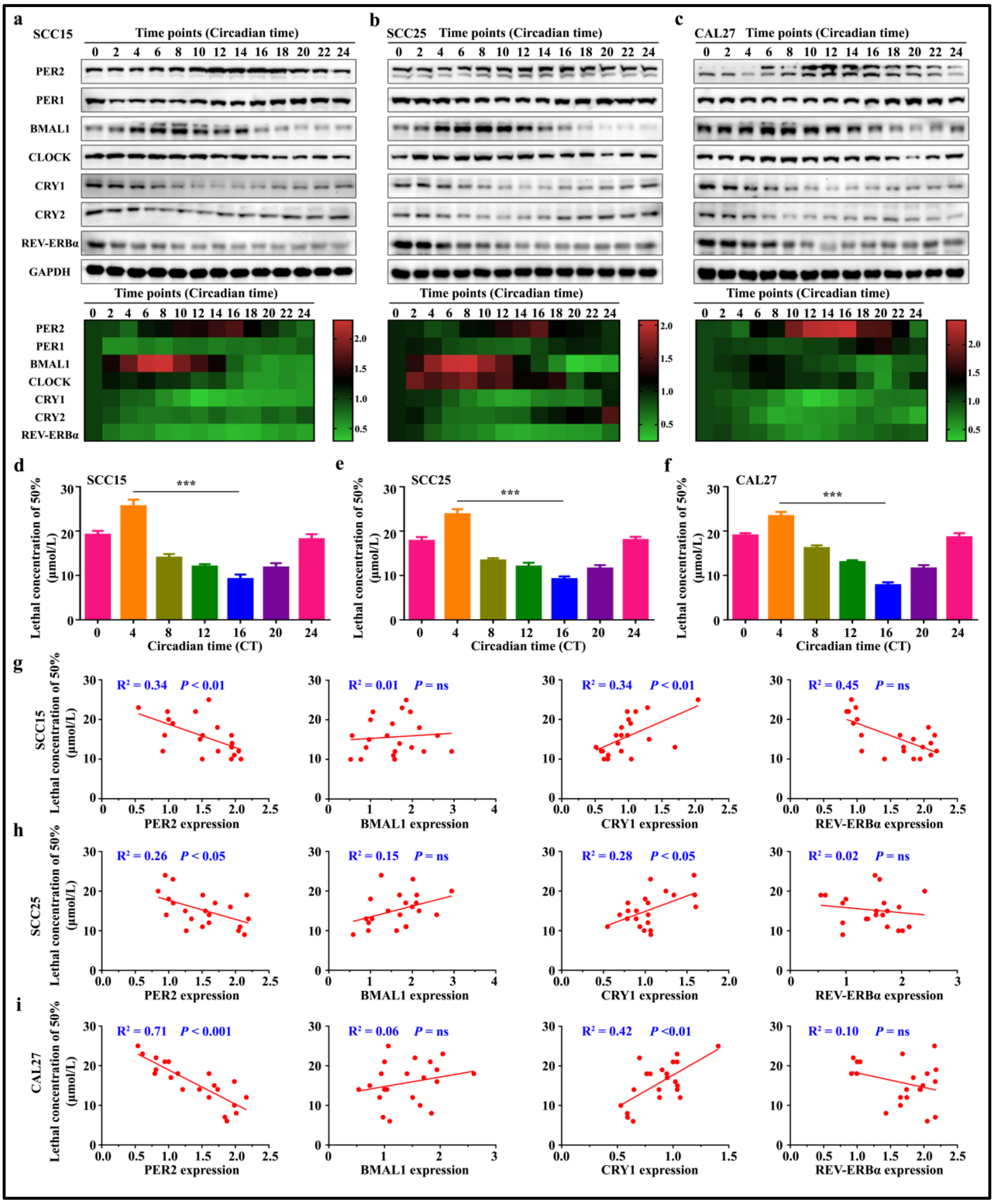

Figure S2. Oxaliplatin chronomodulated efficacy is closely correlated with the diurnal fluctuation of PER2 expression *in vitro*

a-c) Western blot (upper) and densitometric quantification (lower) of PER2, PER1, BMAL1, CLOCK, CRY1, CRY2, REV-ERBα expression levels were measured every two hours over a circadian period in human OSCC cells (n = 3 independent experiments). d-f) The half maximal lethal concentration value (LC_{50}) at indicated time points in human OSCC cells (n = 5 independent experiments). ****P* < 0.001 (compared with ZT4), form ANOVA and Student's

t-test. g-i) The linear correlation was analyzed by coefficient of determination between PER2, BMAL1, CRY1, and REV-ERBα expression levels and lethal concentration of 50% in human OSCC cells.

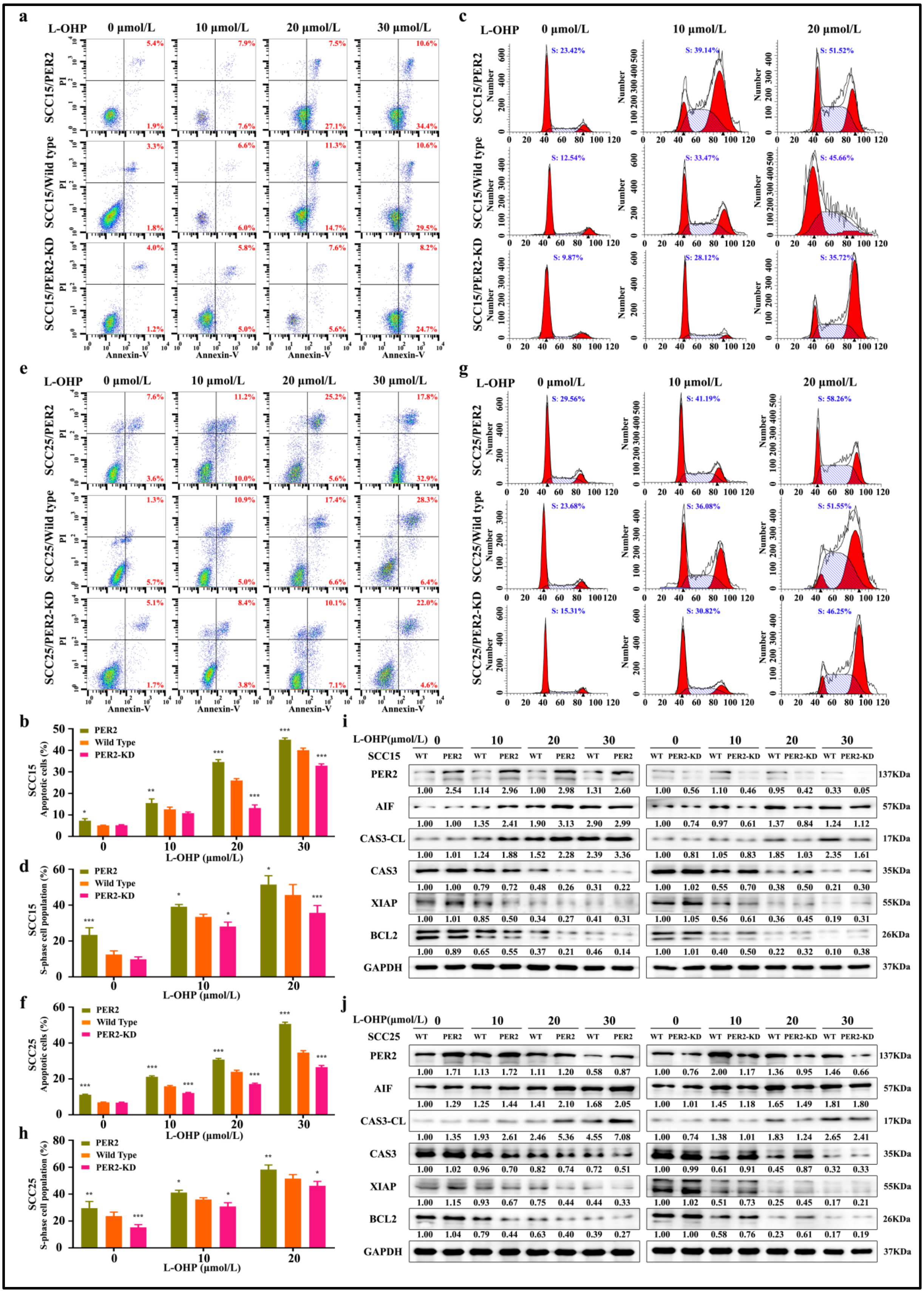

Figure S3. PER2 increases oxaliplatin sensitivity of human OSCC cells

a and b) Apoptosis was evaluated by flow cytometry of PER2-overexpressing or PER2-

knockdown SCC15 cells treated with oxaliplatin (0, 10, 20 or 30 μmol/L, 48-hr). c and d) Cell-cycle phases were determined by flow cytometry of PER2 overexpressing or knockdown SCC15 cells treated with oxaliplatin (0, 10, or 20 μmol/L, 48-hr). e and f) Apoptosis was evaluated by flow cytometry of PER2 overexpressing or knockdown SCC25 cells treated with oxaliplatin (0, 10, 20 or 30 μmol/L, 48-hr). g and h) Cell-cycle phases were determined by flow cytometry of PER2 overexpressing or knockdown SCC25 cells treated with oxaliplatin (0, 10, or 20 μmol/L, 48-hr). i and j) Western blot of PER2, AIF, CAS3-CL, CAS3, XIAP, BCL2, and GAPDH (for loading controls) in PER2 overexpressing or knockdown SCC15 cells (i) or SCC25 cells (j) treated with 0, 10, 20 or 30 μmol/L oxaliplatin. *$P < 0.05$, **$P < 0.01$, and ***$P < 0.001$ (compared with wild type). ANOVA was used. Data represent the mean ± SD of three independent experiments.

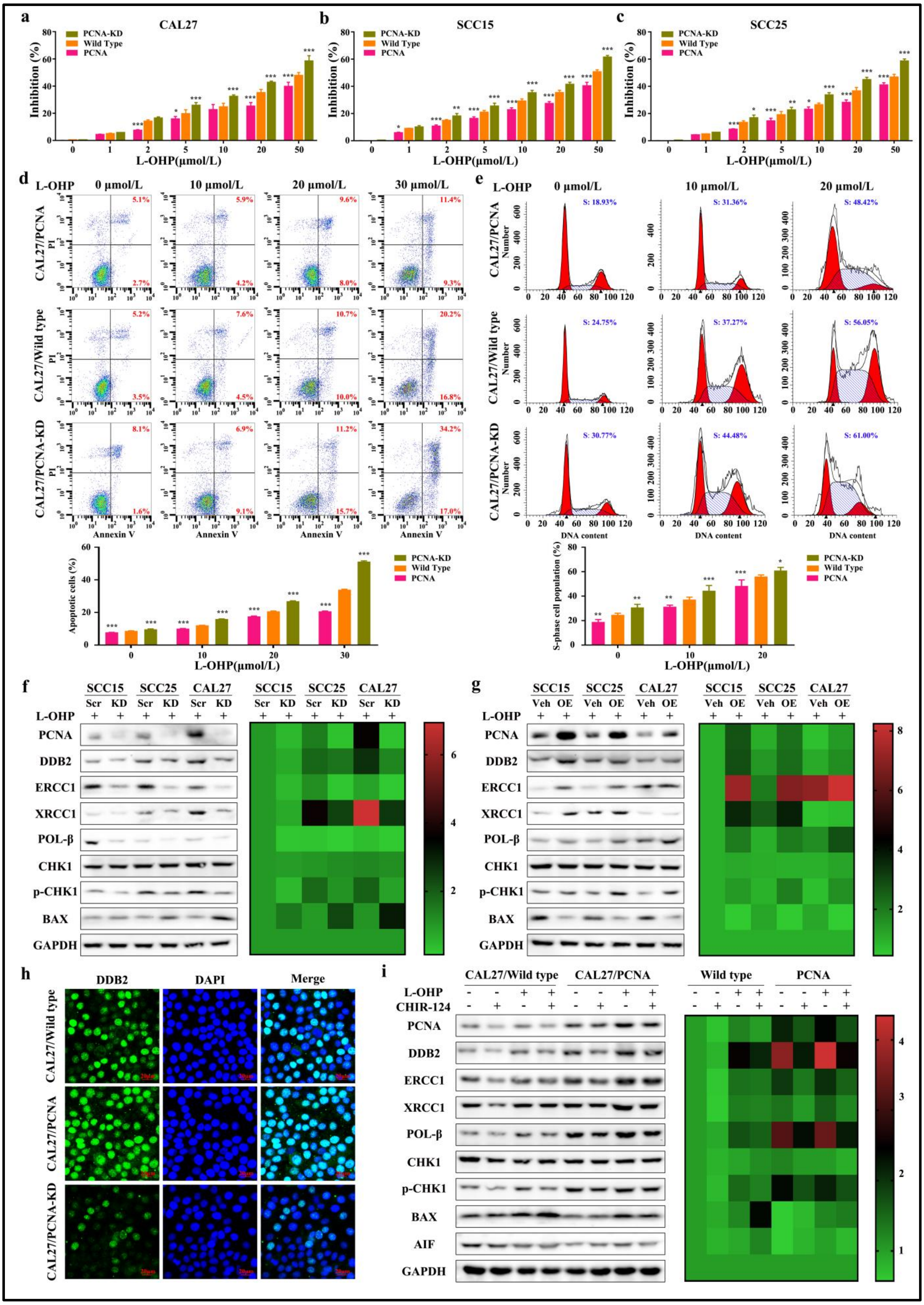

Figure S4. PCNA enhances DNA adducts repair and impairs oxaliplatin sensitivity in human OSCC cells

a-c) Dose-dependent growth inhibition in response to oxaliplatin in PCNA overexpressing or knockdown OSCC cells (n = 5 independent experiments). d) Apoptosis was evaluated by flow cytometry of PCNA overexpressing or knockdown CAL27 cells treated with oxaliplatin (0, 10, 20 or 30 μmol/L, 48-hr). e) Cell-cycle phases were determined by flow cytometry of PCNA overexpressing or knockdown CAL27 cells treated with oxaliplatin (0, 10 or 20 μmol/L, 48-hr). f and g) Western blot and densitometric quantification of the indicated proteins in PCNA knockdown (f) and overexpressing (g) CAL27 cells treated with oxaliplatin (20 μmol/L). GAPDH was used as the loading control. h) Representative confocal images of DDB2 in PCNA overexpressing or knockdown CAL27 cells. Scale bar, 20 μm. i) Western blot and densitometric quantification of the indicated proteins of DNA adducts repair markers in PCNA overexpression or control CAL27 cells treated oxaliplatin (20 μmol/L) with or without CHIR-124 (0.3 nmol/L) (n = 3 independent experiments). GAPDH was used as the loading control. *P < 0.05, **P < 0.01, and ***P < 0.001 (compared with wild type). ANOVA was used. Data represent the mean ± SD of three independent experiments.

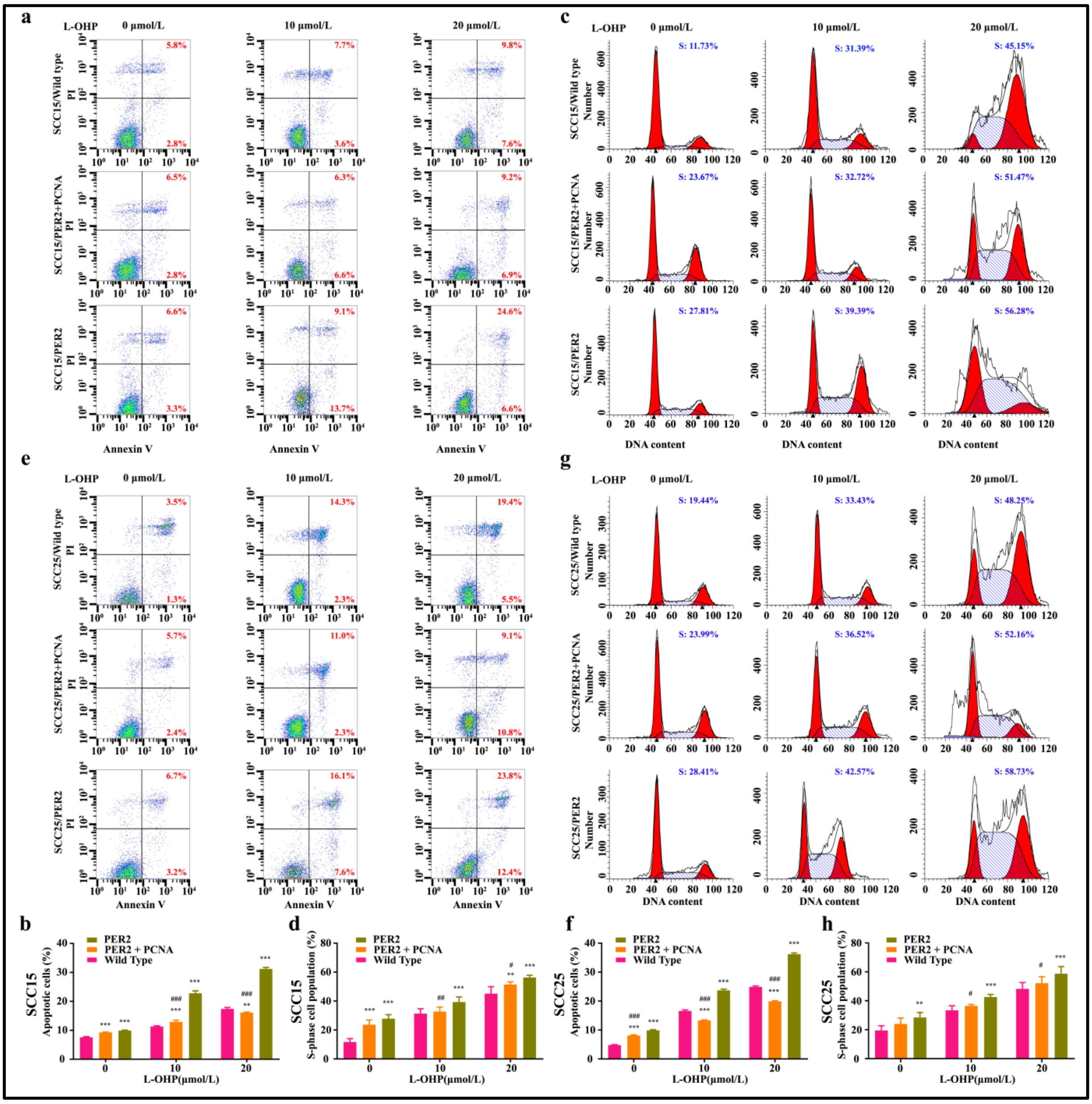

Figure S5. PCNA modulates the functionality of PER2 in oxaliplatin sensitivity

a and b) Apoptosis was evaluated by flow cytometry of PER2-overexpressing or PER2/PCNA double-overexpressing SCC15 cells stained with Annexin V and PI. c and d) Cell-cycle phases were determined by flow cytometry of PER2-overexpressing or PER2/PCNA double-overexpressing SCC15 cells. e and f) Apoptosis was evaluated by flow cytometry of PER2-overexpressing or PER2/PCNA double-overexpressing SCC25 cells stained with Annexin V and PI. g and h) Cell-cycle phases were determined by flow cytometry of PER2-overexpressing or PER2/PCNA double- overexpressing SCC25 cells. $^{**}P < 0.01$, and $^{***}P <$

0.001 (compared with wild type). $^{\#}P < 0.05$, $^{\#\#}P < 0.01$ and $^{\#\#\#}P < 0.001$ (compared with PER2). ANOVA was used. Data represent the mean ± SD of three independent experiments.

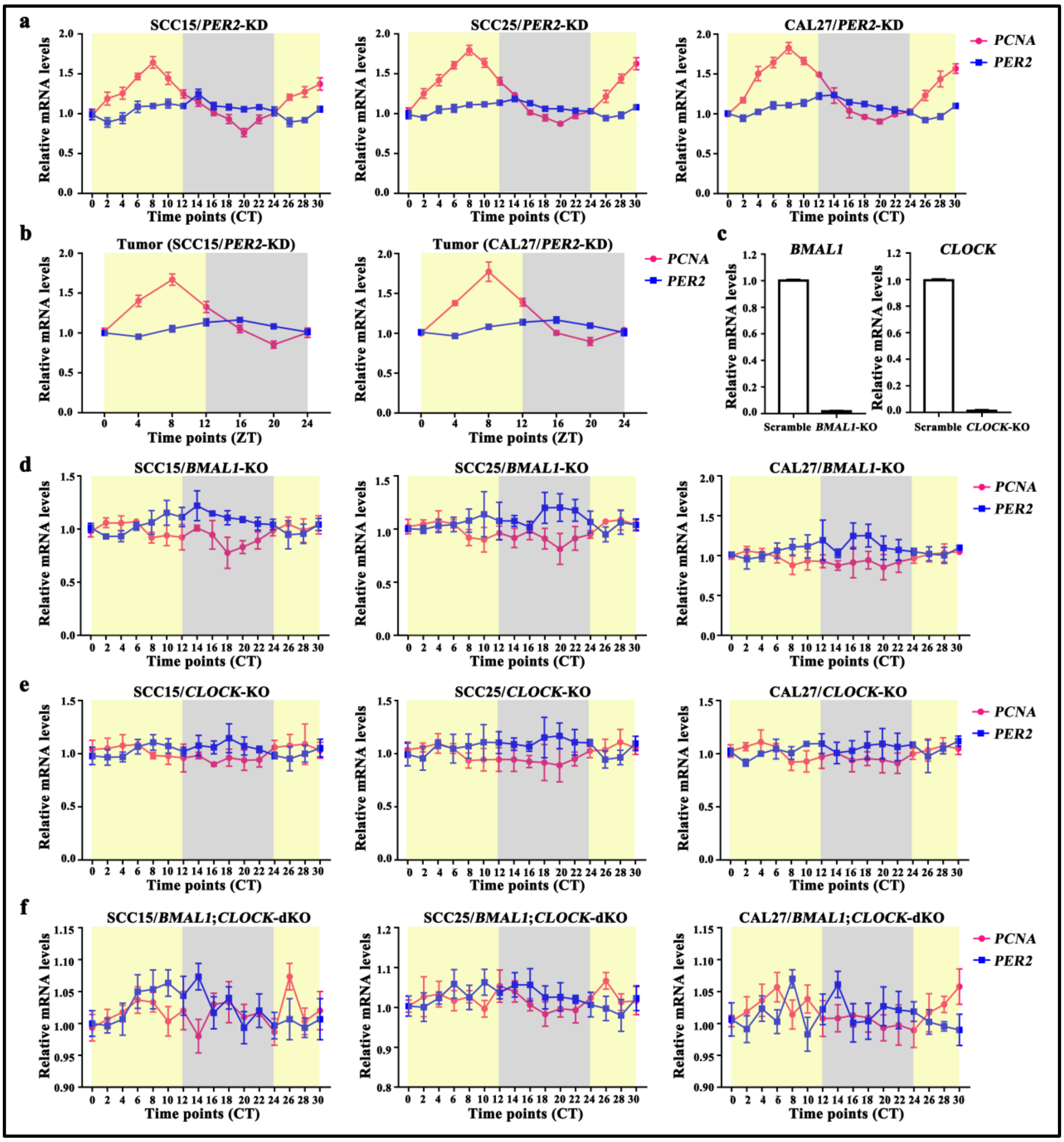

Figure S6. Circadian clock genes regulate the diurnal fluctuation of *PCNA* transcription

a) The mRNA levels of *PCNA* and *PER2* in PER2-knockdown TSCC cells. Samples were collected every 2-hr for 30-hr (n = 3 independent experiments). b) The mRNA levels of *PCNA* and *PER2* in tumors. SCC15/PER2-KD or CAL27/PER2-KD cells were subcutaneously injected into mice, and tumors obtained at indicated time points after four weeks. Samples were collected every 4-hr for 24-hr (n = 3 animals per time point). c) The mRNA levels of *BMAL1* or *CLOCK* in human OSCC cells transfected with BMAL1 or CLOCK knockout (KO) vectors. d) The mRNA levels of *PCNA* and *PER2* in BMAL1-knockout OSCC cells. Samples

were collected every 2-hr for 30-hr (n = 3 independent experiments). e) The mRNA levels of *PCNA* and *PER2* in CLOCK-knockout OSCC cells. Samples were collected every 2-hr for 30-hr (n = 3 independent experiments). f) The mRNA levels of *PCNA* and *PER2* in BMAL1/CLOCK double-knockout OSCC cells. Samples were collected every 2-hr for 30-hr (n = 3 independent experiments).

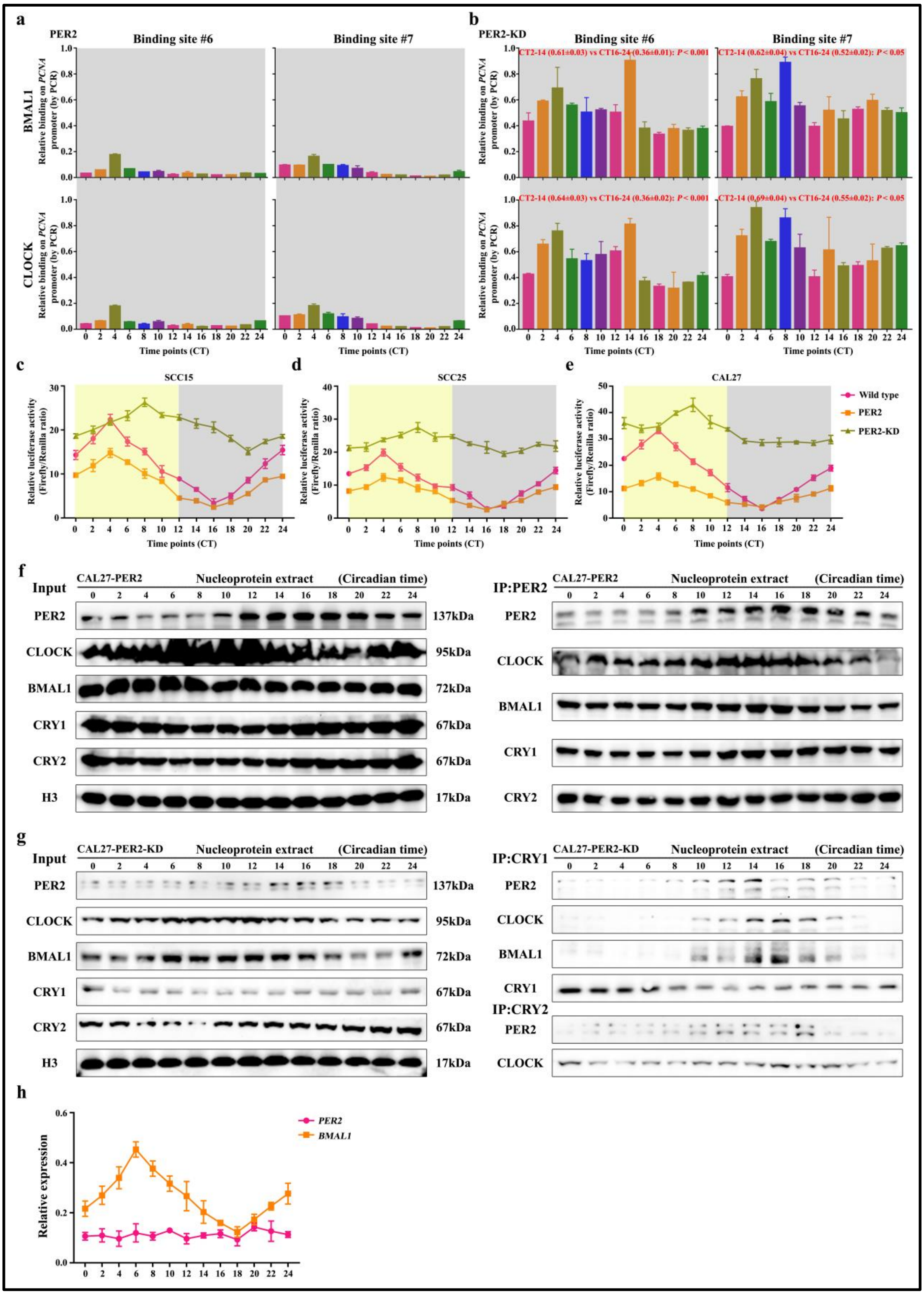

Figure S7. Overexpressed PER2 suppresses *PCNA* transcription through removing CLOCK-BMAL1 heterodimers from its promoter extensively

a and b) CLOCK and BMAL1 bound to the *PCNA* promoter at predicted binding site #6 and #7 in CAL27/PER2 cells (a) and CAL27/PER2-KD cells (b) at indicated time points.

Chromatin immunoprecipitation assay was performed using anti-BMAL1 or anti-CLOCK antibodies with anti-IgG antibody as a negative control. c-e) Diurnal luciferase reporter assays were performed to measure the transcriptional activities of wild-type *PCNA* promoter in human OSCC cells with PER2 overexpression or PER2 knockdown at indicated time points. f) Coimmunoprecipitation (Co-IP) assay was performed in nucleoprotein extracts obtained across a circadian cycle with an anti-PER2 antibody, or IgG (served as a negative control) and detected by western blot analysis with anti-PER2, anti-CLOCK, anti-BMAL1, anti-CRY1 or anti-CRY2 antibodies in CAL27/PER2 cells. g) Co-IP was performed in nucleoprotein extracts obtained across a circadian cycle with an anti-CRY1 antibody, anti-CRY2 antibody, or IgG (served as a negative control) and detected by western blot analysis with anti-PER2, anti-CLOCK, anti-BMAL1, anti-CRY1 or anti-CRY2 antibodies. h) qRT-PCR was performed to compare the levels of *BMAL1* and *PER2* at intervals of two hours in PER2 overexpressed CAL27 cells. Values were expressed as relative expression levels to housekeeping gene *GAPDH*. Data represent the mean ± SD of three independent experiments.

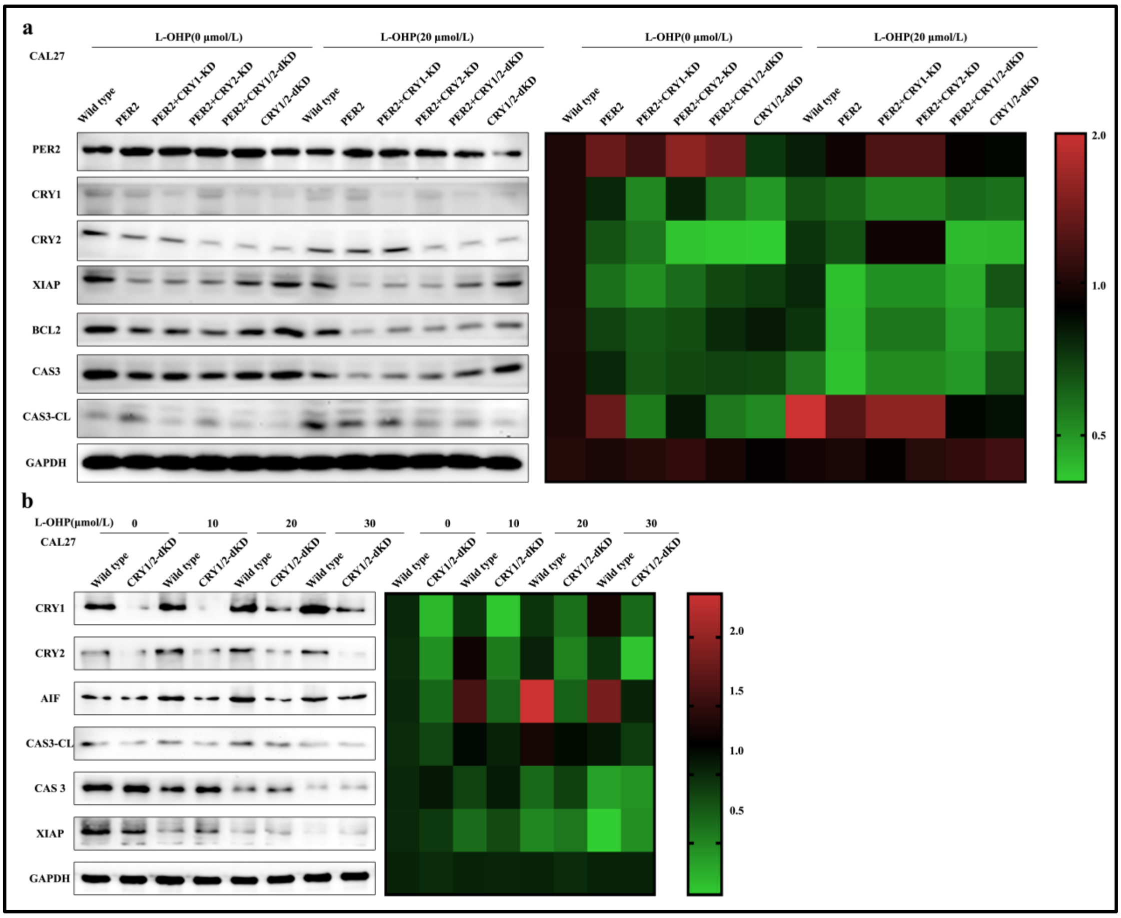

Figure S8. CRYs correlate with PER2 in oxaliplatin sensitivity modulation

a) Western blot of PER2, CRY1, CRY2, XIAP, BCL2, CAS3, CAS3-CL, and GAPDH (for loading controls) in CAL27 cells transfected with vectors (PER2-overexpression, CRY1-knockdown, CRY2-knockdown or CRY1/2-double knockdown) and treated with 0 or 20 μmol/L oxaliplatin. b) Western blot of CRY1, CRY2, AIF, CAS3-CL, CAS3, XIAP, and GAPDH (for loading controls) in CRY1/2 double-knockdown CAL27 cells treated with 0, 10, 20 or 30 μmol/L oxaliplatin. Data represent the mean ± SD of three independent experiments.

Supplementary Tables

Table S1. Primer sequences used in qRT-PCR and ChIP assays

LOCUS	FORWARD (5'-3')	REVERSE (5'-3')
qRT-PCR		
h-DDB2	TGACAGGAGGGCTACATCCTTG	GAAGGTGGGTTTGTCCTTGATGC
h-ERCC1	CGGGGCAAAATCCAACAGCA	AGTCGGGAATTACGTCGCCA
h-XRCC1	AAGGCAGGCGAGAAGACCAT	AGCACCTCCACGAAAGCTGA
h-POL-β	TGACCCATCCCAGCTTCACTTC	ACCCATGAACTTTGTCTCACCCTT
h-PCNA	AGCCACTCCACTCTCTTCAACG	TTCATCCTCGATCTTGGGAGCC
h-RFC3	CCCTGCTTATGTGTGAAGCCTG	GCCTTTGTGGAGTTTGCTGACTG
h-TNFSF10	GAGCTGAAGCAGATGCAGGACA	TGACGGAGTTGCCACTTGACTT
h-TP53	TGCGTGTTTGTGCCTGTCCT	GTGGTTTCTTCTTTGGCTGGGG
h-ENDOG	ACGACACGTTCTACCTGAGCA	AGTGGCCCTGTGCAGACATA
h-CYCS	GGCCCCTGGATACTCTTACACA	GCTATTAAGTCTGCCCTTTCTTCC
h-TNFRSF10C	AAGTTCCTGCACCATGACCAGA	TACTGACTTGGACTTCCCCACT
h-PRKAR1B	TGACCAACATCAGCGAGGGA	AGCGTGCTGCCCATAAGGAT
h-DBF4	CACAAGCTACACAGAAAAGAGTGG	TGAAGGGGACGAGAAAAACGC
h-E2F1	AGGAGTTCATCAGCCTTTCCCC	GGTCCCCAAAGTCACAGTCGAA
h-E2F2	CTGCTACCTACTACACACCGCT	CCCTCCAGATCCAGCTTCCTTT
h-CCND1	GCTGTGCATCTACACCGACAAC	CAATGAAATCGTGCGGGGTCA
h-MAD2L1	CAGCTACGGTGACATTTCTGCC	TGGTCCCGACTCTTCCCATTT
h-CCNE2	GGCCAAGAAGAGGAAAACTACCC	TGATAATGCAAGGACTGATCCCCC
h-FBXL3	GGTGGACAGCAGCAAGGAATCA	AAGCGAAGACAGGGATTTGGAGT
h-PER1	TGAGTCCAACGGGCATGAGT	AGGCAATGGAACTGCTGGGT
h-PER2	TTCACCACATCCTGGAACAAAC	TTCACCACATCCTGGAACAAAC
h-PER3	AAGGCGGAGCAGATGACCTT	CCATTCGGTTCTGTGCGTGT
h-RORB	TGGCAGACCCACACCTATGAA	CACCACGTATTGGATGGCGT
h-BMAL1	GGATGTGACCGAGGGAAGAT	CGTCGTGCTCCAGAACATAAT
m-PER2	CACACTTGCCTCCGAAATAACTC	AGCGCACGGCTGTCTGA
ChIP		
Promoter1	CTCCAGCCAACCATTGAG	ATGAGTGACCTGTAGATGTT
Promoter2	GCTCTGTCATCCTGGGAA	GCTGCTAAGGGAACTGTG
Promoter3	CTCACAGTTCCCTTAGCA	CGCCATTGGTGGATATTC
Promoter4	CCTCCTTCCTACTCTCCTC	CCATAGCATTTGCCTCCT
Promoter5	AAGGCAGGCAGATCACAT	TCTTGGCTAACCGCAATC
Promoter6	AATCCTGTCCATCCTGTAAC	GGTTCAAGTGAGTCTCCTG
Promoter7	CTAAGTGCTCAAAGGTGTTT	GCTCCCTATCATCCTCTTAA
Promoter8	GGAATGTTAAGAGGATGATAGG	CGAAAGTGAAAGTGAAATGC

Table S2. Antibodies used in this study

Rabbit monoclonal anti-KAT13D/CLOCK antibody	Abcam	Cat#ab134165
Rabbit polyclonal anti-KAT13D/CLOCK antibody	Abcam	Cat#ab3517
Mouse monoclonal anti-CLOCK Antibody (C-8)	Santa Cruz	Cat#sc-271603
Rabbit polyclonal anti-BMAL1 antibody	Abcam	Cat#ab93806
Rabbit polyclonal anti-BMAL1 antibody	Abcam	Cat#ab3350
Mouse monoclonal anti-BMAL1 Antibody (A-6)	Santa Cruz	Cat#sc-373955
Rabbit polyclonal anti-PER1 antibody	Abcam	Cat#ab3443
Rabbit monoclonal anti-PER2 antibody	Abcam	Cat#ab179813
Mouse monoclonal anti-PER2 antibody	Santa Cruz	Cat#sc-377290
Rabbit polyclonal anti-PER2 antibody	Santa Cruz	Cat#sc-25363
Rabbit polyclonal anti-CRY1 antibody	Abcam	Cat#ab104736
Mouse monoclonal anti-CRY1 antibody	Abcam	Cat#ab54649
Rabbit polyclonal anti-CRY1 antibody	Abcam	Cat#ab245564
Rabbit polyclonal anti-CRY2 antibody	Abcam	Cat#ab38872
Rabbit polyclonal anti-CRY2 antibody	Abcam	Cat#ab93802
Rabbit polyclonal anti-REV-ERBα antibody	Proteintech	Cat#14506-1-AP
Rabbit polyclonal anti-GAPDH antibody	Proteintech	Cat#10494-1-AP
Rabbit polyclonal anti-AIF antibody	Proteintech	Cat#17984-1-AP
Rabbit polyclonal anti-Caspase 3 antibody	Cell Signaling Technology	Cat#9662
Rabbit polyclonal anti-XIAP antibody	Abcam	Cat#ab21278
Mouse monoclonal anti-BCL2 antibody	Proteintech	Cat#60178-1-lg
Rabbit monoclonal anti-DDB2 antibody	Abcam	Cat#ab181136
Mouse monoclonal anti-DDB2 antibody for IF	Abcam	Cat#ab51017
Rabbit monoclonal anti-ERCC1 antibody	Abcam	Cat#ab129267
Rabbit monoclonal anti-XRCC1 antibody	Abcam	Cat#ab134056
Rabbit monoclonal anti-DNA polymerase beta antibody	Abcam	Cat#ab175197
Rabbit monoclonal anti-Chk1 antibody	Abcam	Cat#ab32531
Rabbit monoclonal anti-phospho-Chk1 (S345)	Cell Signaling Technology	Cat#2348
Rabbit Polyclonal anti-BAX antibody	Proteintech	Cat#50599-2-Ig
Rabbit monoclonal anti-PCNA antibody	Abcam	Cat#ab92552
Mouse monoclonal anti-PCNA antibody for IHC	Abcam	Cat#ab29
Rabbit Polyclonal anti-Cytokeratin 19 antibody	Proteintech	Cat#10712-1-AP
Rabbit Polyclonal anti-Vimentin antibody	Proteintech	Cat#10366-1-AP
Rabbit Polyclonal anti-Histone H3 antibody	Proteintech	Cat#17168-1-AP
HRP-conjugated Affinipure Goat Anti-Rabbit IgG	Proteintech	Cat#SA00001-2
HRP-conjugated Affinipure Goat Anti-Mouse IgG	Proteintech	Cat#SA00001-1
Goat polyclonal anti-mouse IgG, Alexa Fluor 488-conjugated	Abcam	Cat#ab150117
Goat polyclonal anti-rabbit IgG, Alexa Fluor 488-conjugated	Abcam	Cat#ab150077
